# METTL14 suppresses proliferation and metastasis of colorectal cancer by down-regulating oncogenic long non-coding RNA *XIST*

**DOI:** 10.1186/s12943-020-1146-4

**Published:** 2020-02-28

**Authors:** Xiao Yang, Sen Zhang, Changyu He, Pei Xue, Luyang Zhang, Zirui He, Lu Zang, Bo Feng, Jing Sun, Minhua Zheng

**Affiliations:** 1 Division of Gastrointestinal and Colorectal Surgery, Ruijin Hospital, Department of General Surgery, Shanghai Jiao Tong University, School of Medicine, Shanghai, 200001 China; 2 Shanghai Institute of Minimally Invasive Surgery, Ruijin Hospital, Shanghai Jiao Tong University, School of Medicine, Shanghai, 200001 China

**Keywords:** m6A, METTL14, Long non-coding RNA, Colorectal cancer, RNA epigenetics

## Abstract

**Background:**

N6-methyladenosine (m6A) is the most prevalent RNA epigenetic regulation in eukaryotic cells. However, understanding of m6A in colorectal cancer (CRC) is very limited. We designed this study to investigate the role of m6A in CRC.

**Methods:**

Expression level of METTL14 was extracted from public database and tissue array to investigate the clinical relevance of METTL14 in CRC. Next, gain/loss of function experiment was used to define the role of METTL14 in the progression of CRC. Moreover, transcriptomic sequencing (RNA-seq) was applied to screen the potential targets of METTL14. The specific binding between METTL14 and presumed target was verified by RNA pull-down and RNA immunoprecipitation (RIP) assay. Furthermore, rescue experiment and methylated RNA immunoprecipitation (Me-RIP) were performed to uncover the mechanism.

**Results:**

Clinically, loss of METTL14 correlated with unfavorable prognosis of CRC patients. Functionally, knockdown of METTL14 drastically enhanced proliferative and invasive ability of CRC cells in vitro and promoted tumorigenicity and metastasis in vivo. Mechanically, RNA-seq and Me-RIP identified lncRNA *XIST* as the downstream target of METTL14. Knockdown of METTL14 substantially abolished m6A level of *XIST* and augmented *XIST* expression. Moreover, we found that m6A-methylated *XIST* was recognized by YTHDF2, a m6A reader protein, to mediate the degradation of *XIST*. Consistently, *XIST* expression negatively correlated with METTL14 and YTHDF2 in CRC tissues.

**Conclusion:**

Our findings highlight the function and prognostic value of METTL14 in CRC and extend the understanding of the importance of RNA epigenetics in cancer biology.

## Background

Colorectal cancer (CRC) continues to be a severe health problem worldwide, resulting in 700,000 deaths annually [1]. The mortality of CRC is primarily due to post-operational recurrence and metastasis [2]. Despite of recent advances in therapeutic strategies, including modified surgical techniques and improved adjuvant therapy, the prognosis of CRC is still far from satisfactory [3]. Thus, understanding the molecular mechanisms of CRC carcinogenesis and progression is essential to future diagnostic and therapeutic inventions.

Traditionally, epigenetic regulation refers to chemical modifications on DNA or histones, which regulates gene expression independent of genome sequences alterations [4]. Dysregulation of epigenetic modifying enzymes profoundly contributes to human diseases and has been frequently reported in multiple types of cancer [5]. Similarly, RNAs also carry hundreds of various sites for distinct post-transcriptional modifications. N6-methyladenosine (m6A) is the most predominant modification of mRNA in eukaryotic cells [6, 7]. It is a reversible chemical process dynamically controlled by the balanced activities of m6A methyltransferases and demethylases. Since m6A modification has been demonstrated to play a vital role in RNA translation, stability and alternative splicing, perturbations of m6A components are associated with human diseases, especially with cancers [8–10]. Dysregulation of m6A “writer” protein, methyltransferase-like 3 (METTL3) and methyltransferase-like 14 (METTL14), has been reported in liver cancer [11, 12], lung cancer [9] and glioblastoma [13]. Besides, the m6A “eraser”, fat mass- and obesity-associated protein (FTO), was found to act as oncogene in acute myeloid leukemia [14] and lung squamous cell carcinoma [15]. However, the status of m6A and the underlying mechanisms in CRC still remain largely unknown.

X inactivate-specific transcript (*XIST*) is a newly identified lncRNA which serves as an oncogene in several types of solid tumors including lung cancer [16], ovarian cancer [17], liver cancer [18], and colorectal cancer [19]. The molecular mechanism governing the action of this lncRNA involves its activating effects on a variety of tumorigenic signal pathways. For instance, *XIST* was shown to expedite proliferation and metastasis of CRC via sequestering miR-200b and promoting epithelial–mesenchymal transition [19]. Importantly, empowered by individual-nucleotide resolution UV crosslinking and immunoprecipitation (iCLIP) method, recent m6A mapping study has identified at least 78 m6A residues distributed along *XIST* [20]. Moreover, m6A methylation on special sites is indispensable for *XIST* to induce transcriptional silencing of genes on the X chromosome [20]. These results imply that m6A may play a crucial role in the regulation of lncRNAs, which reminds us to explore its potential effects on human cancers, especially on CRC.

In this study, we investigated the role of METTL14, a major m6A “writer”, in CRC and addressed the underlying mechanism. We found that METTL14 suppressed proliferation and invasion of CRC cells by down-regulating oncogenic lncRNA *XIST* in a m6A dependent manner. In line with this, knockdown of METTL14 in CRC cells resulted in decreased m6A-methylation levels of *XIST*, leading to elevated *XIST* levels and enhanced tumor driving effects. In total, we highlight the important role of METTL14 and m6A in colorectal cancer and provide a promising marker for predicting prognosis of CRC.

## Methods

### Patients and samples

Tumor tissues and adjacent normal tissues (> 5 cm away from tumor) were collected from 37 CRC patients undergoing radical surgery in our institute. Clinical parameters including age, sex, stage, pathological diagnosis, TNM status and recurrence were also collected. No patient had received local or systemic treatment before operation. Fresh tumor and paired normal tissues were frozen in liquid nitrogen and stored at − 80 °C. The rest part of specimens was fixed in the formalin and embedded in paraffin for pathological and immunohistochemical (IHC) analysis. Our study protocol has been approved by the Ethics Committee of Ruijin Hospital, Shanghai Jiao Tong University, School of Medicine. Informed consent was obtained from each enrolled patient.

### IHC analysis

Tissue sections were deparaffinized, rehydrated, and microwaved-heated in sodium citrate buffer (10 mmol/L, pH 6.0) for antigen retrieval. Then, the slides were incubated with primary antibody. Then the expression levels of target proteins in tissue were examined by two independent pathologists blinded to the clinical characteristics of the patients according to proportion of cell staining (0 = 0%, 1 = ≤ 25%, 2 = 26 to 50%, 3 = 51 to 75%, 4 = > 75% positive cells) and the staining intensity (0 = no staining, 1 = weak, 2 = moderate, 3 = strong). A final score was calculated by multiplying the above two scores. Protein expression was considered high if the final score was greater than 6 points and low if the final score was 6 points or less.

### Cell culture and transfection

Human colon epithelial cell line NCM460 was purchased from the Cell Bank of Type Culture Collection of the Chinese Academy of Sciences (Shanghai, China). Five CRC cell lines (SW480, SW620, HCT116, LoVo, and HT29) were previously purchased from the American Type Culture Collection (ATCC, Manassas, VA, USA) and preserved in the research institute of general surgery. SW480 and NCM460 cells were cultured in RPMI-1640 medium. HT29 and HCT116 were cultured in McCoy’s 5A medium, SW620 cells were cultured in Leibovitz’s L-15 medium. LoVo was cultured in Ham’s F-12 K (Kaighn’s) Medium. All kinds of medium were purchased from Invitrogen (Carlsbad, CA, USA) and supplemented with 10% fetal bovine serum (FBS, HyClone, Logan, USA), 100 U/ml penicillin and 100 μg/ml streptomycin. Cells were maintained in a 37 °C incubator with 5% CO_2_. The full length METTL14 cDNA was synthesized by RT-PCR from normal colon epithelial cells (NCM460) and then sub-cloned into the pcDNA3.1 vector to construct pcDNA-METTL14 overexpression (METTL14-OE) plasmid. The shRNA sequences targeting METTL14 were synthesized with a well-established annealing method and then cloned into pLKO.1 plasmid (Sigma Aldrich, St. Louis, USA). SiRNAs (siMETTL14, siXIST, siWTAP) and negative control were purchased from HanYin Bio-Tech Co.,Ltd. (Shanghai, China). For transient transfection, cells were transfected with plasmids encoding target sequences or siRNAs using Lipofectamine 3000 reagent (Invitrogen) according to the manufacturer’s instructions. Stable clones were selected by puromycin (1 μg/ml). All transfects were tested regularly by western blot to ensure the efficiency of over-expression or knockdown. Details of sequences or hairpin sequences used in this study were listed in Additional file 2: Table S2.

### Cell proliferation and invasion assay

Cell viability was measured with cell count assay and colony formation assay. For cell count assay, control and transfected cells were cultured in a 96-well plate (3000 cells/well). Triplicate wells were measured in each group. Cell viability was determined every 24 h. The plate was incubated at 37 °C for 2 h after each well was added with 10 μl CCK-8 solution. Then the spectrophotometric absorbance was measured at 450 nm for each sample. For the colony formation assay, a certain number of control or transfected cells were planted into a six-well plate and maintained in culture media containing 10% FBS for 2 weeks. Colonies were fixed with methanol and stained with crystal violet (Sigma-Aldrich). The colony number was determined by counting stained colonies using ImageJ software. Transwell invasion assay were performed as we described elsewhere. Briefly, 8 × 10^4^ cells in 200 μL serum-free media were seeded in the top chamber (8.0 μm pore size, Corning, USA) with membrane coated by Matrigel (BD Bioscience, USA). 600 μL of 10% FBS-containing medium was placed into the bottom chamber as an attractant. After incubation for 24 h, the cells that did not invade to the lower side of the chamber were removed from the top side. The chambers were then stained with crystal violet and photographed.

### Western blot

Briefly, cells were washed with PBS and lysed with RIPA buffer (Thermo Scientific, MA, USA). The lysates were then sonicated on ice and centrifuged at 14,000 g for 5 min at 4 °C to remove cell debris. Next, the supernatants were resolved in 12.5% SDS-PAGE (30 μg/lane) and transferred onto 0.22-lm polyvinylidene fluoride (PVDF) membranes (Millipore, MA, USA). The membranes were probed with proper antibodies overnight at 4 °C. After washing for three times, the membrane was incubated in HRP-labeled secondary antibody for 2 h at room temperature. Protein bands were revealed by enhanced chemiluminescence (ECL) method. The following antibodies used in this study were purchased from Cell Signaling Technology (Beverly, MA, USA): rabbit anti-METTL14 (D8K8W), rabbit anti-WTAP (56501), rabbit anti-GAPDH (D6H11).

### RNA extraction and real-time PCR

Total RNA was extracted using TRIzol reagent (Ambion) and cDNA synthesis was performed using a reverse transcription kit (Promega, Madison, USA) according to the manufacturer’s instructions. PCR was conducted using the SYBR Green Master Mix (Applied Biosystems, MA, USA) and the Applied Biosystems 7900HT sequence detection system. After the reactions were complete, relative gene expression level was calculated using the 2^−ΔΔCt^ method. GAPDH was used as an endogenous control. Primers used were as follows: METTL14: sense: 5′-GAACACAGAGCTTAAATCCCCA-3′; antisense: 5′-TGTCAGCTAAACCTACATCCCTG-3′. *XIST*: sense: 5′-GCATAACTCGGCTTAGGGCT-3′, antisense: 5′-TCCTCTGCCTGACCTGCTAT-3′.

### RNA immunoprecipitation

Cells cultured in 10 cm plate was washed twice with ice-cold PBS and scraped off in 1 mL PBS. Then the cell was centrifuged and re-suspended in an equal pellet volume of complete RIP lysis buffer (Merck Millipore). 5 μg antibody was pre-bound to Protein A/G magnetic beads in immunoprecipitation buffer (20 mM Tris-HCl pH 7.5, 140 mM NaCl, 0.05% TritonX-100) for 2 h and then incubated with 100 μl cell lysates over night at 4 °C with rotation. Then RNA was eluted from the beads by incubating with 400 μl elution buffer for 2 h. The eluted RNA was precipitated with ethanol and dissolved with RNase-free water. Enrichment of certain fragments was determined by real-time PCR. Primer used for *XIST* quantification was designed as follows: sense: 5′-CTGCTGCAGCCATATTTCTTAC-3′, anti-sense: 5′-TACGCCATA AAGGGTGTTGG-3′. IgG was used for negative control. For m6A-RNA immunoprecipitation (Me-RIP), *XIST* extracted from equal amount cell lysates was used as input to measure the m6A-methylated rate of *XIST*. Antibodies used in this experiment were as follows: anti-m6A (ab190886, Abcam), anti-YTHDF1 (ab99080, Abcam), anti-YTHDF2 (ab170118, Abcam), anti-YTHDF3 (ab103328, Abcam), anti-YTHDC1 (ab122340, Abcam), anti-YTHDC2 (ab176846, Abcam). Anti-IgG (Cell Signaling Technology, #2729).

### RNA-binding protein pull-down assay

RNA pull-down assay was performed with RNA-Protein Pull-Down Kit (Pierce, USA) according to the protocol of manufacturer. Briefly, full length of *XIST* was transcribed in vitro using Large Scale RNA Production Systems (Promega, USA) and labeled with Biotin using Biotin RNA Labeling Mix (Roche, Switzerland). Then 1 mg cell lysates extracted from CRC cells was incubated with 3 μg purified biotinylated transcripts for 1 h at 4 °C with rotation. Then the streptavidin agarose beads were added into cell protein lysate to precipitate the RNA-protein complex. The beads were washed three times and boiled in sodium dodecyl sulfate (SDS) buffer to retrieve proteins for western blot analysis.

### RNA-seq and analyses

For RNA sequencing, purified RNA from shMETTL14 and control cells was used for library construction with Illumina TruSeq RNA Sample Prep Kit (FC-122-1001) and then sequenced with Illumina HiSeq 2000. Raw reads were aligned to the human genome GRCh37/hg19 by Bowtie2. Differentially expressed genes (DEGs) between treatment and control samples were identified with limma-voom method. A heatmap clustered by k-means was used to show DEGs or transcripts.

### Animal experiments

Establishment and analysis of nude mice (male BALB/c nu/nu nude mice, 4-week-old) subcutaneous xenograft model was performed as we described before [21]. For liver metastasis model, mice were anaesthetized and an incision was made through the skin and peritoneum to expose the spleen. 1 × 10^6^ HCT116 cells were injected into the spleen (*n* = 4 each group). All mice were kept until death due to the neoplastic progression or until the end of the experiment (6 weeks). The mice livers were removed and carefully dissected to evaluate the metastatic lesions. Animal experiments were all carried out according to the Guide for the Care and Use Laboratory Animals of Ruijin Hospital, Shanghai Jiao Tong University School of Medicine.

### Statistical analysis

R software (version 3.5.1) was used for statistical analysis and data visualization. Protein and RNA levels were compared using two-tailed Student’s *t* test. Correlations between METTL14 expression in CRC tissues and clinicopathological features were analyzed with Pearson Chi-square (χ2) test. Overall survival (OS) and recurrence free survival (RFS) was assessed by Kaplan-Meier method, difference between survival curves was determined by log-rank test. Differences with a *p* value < 0.05 were considered as statistically significant.

## Results

### METTL14 was downregulated in CRC

**Fig. 1:**
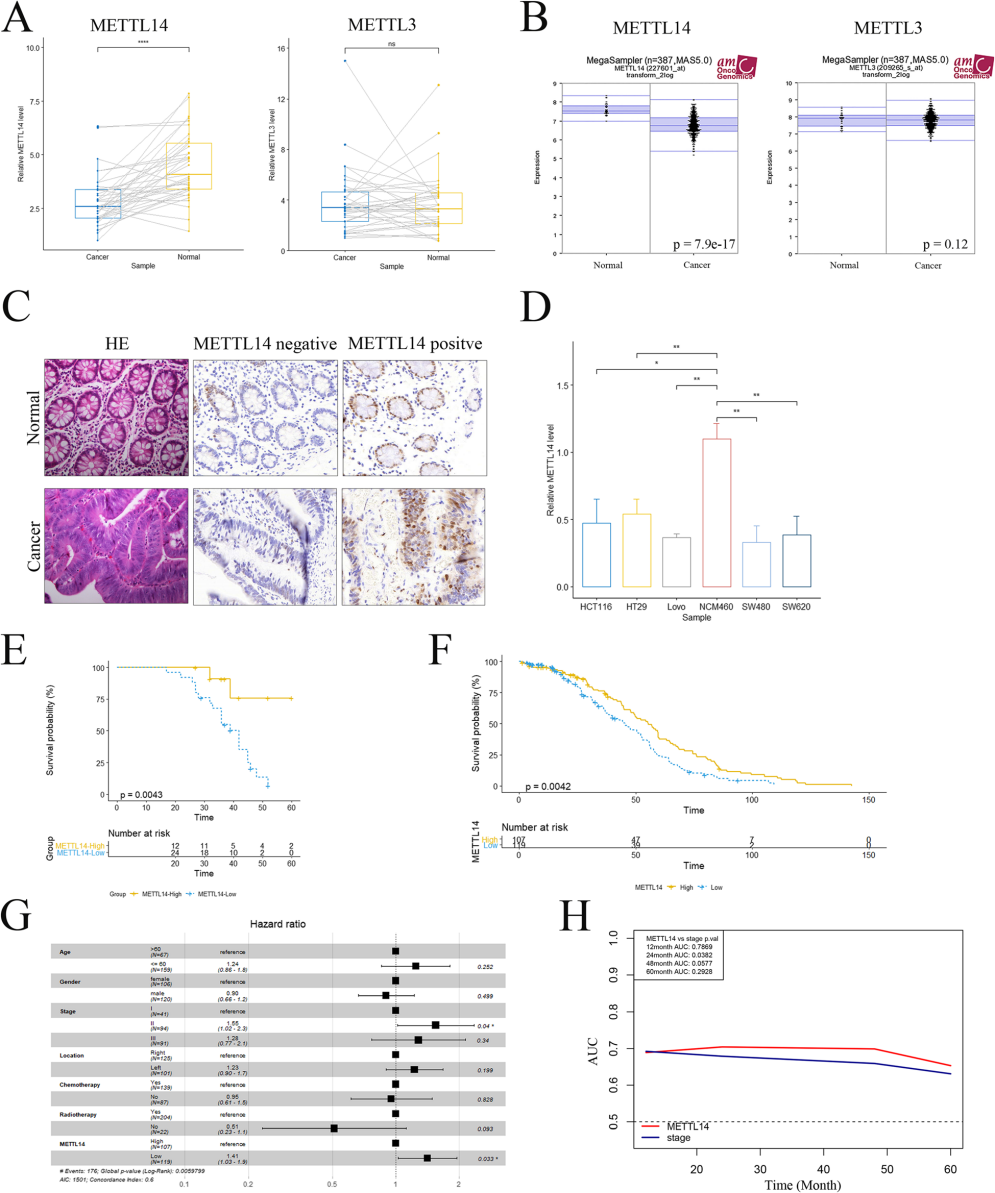
METTL14 was down-regulated in human colorectal cancer***.*a** Real-time PCR analysis of *METTL14* and *METTL3* expression levels in colorectal cancer and paired normal tissue. ns, not significant. **, *p* < 0.01. **b** Mega-sampler analysis of expression patterns of *METTL14* and *METTL3* in cancer and normal tissues from GEO dataset (GSE14333). *P* values were as indicated. **c** Representative images of immunohistochemical (IHC) staining of METTL14 in colorectal cancer and paired normal tissue. **d** Expression levels of *METTL14* in different CRC cell lines and normal NCM460 cells. **, *p* < 0.01. **e-f** Kaplan-Meier analysis of RFS of CRC patients from our cohort (**e**) and GSE14333 dataset (**f**). **g** Multivariate analysis of clinical prognostic parameters for RFS of CRC patients in GSE14333 cohort. *P* values were as indicated. **h** Time-dependent ROC analysis showing the predictive ability of METTL14 of RFS of CRC patients. P values were as indicated. PCR was analyzed by paired student’s *t*-test (**a**) and nonpaired student’s *t*-test (**d**). Mega-sampler analysis of GEO data (**b**) was generated by R2 online tools (http://r2.amc.nl/). Difference of RFS (**e** and **f**) between two subgroups was analyzed with Log-rank test. Time-dependent ROC analysis was constructed with Cox model from ‘Survival’ R package

To investigate the potential role of m6A in colorectal cancer, we first detected the mRNA levels of major m6A methyltransferase including METTL3 and METTL14 in 37 CRC and paired normal samples. As shown, METTL14 was remarkably down-regulated in cancerous compared to paired normal samples (Fig. 1a, left panel). In contrast, no significant difference was observed in the expression level of METTL3 (Fig. 1a, right panel). Moreover, similar expression pattern of METTL14 and METTL3 (Fig. 1b) in CRC were validated in an expanded cohort containing 387 cases of CRC patients from Gene Express Omnibus (GEO) dataset (GSE14333), which further supported our initial findings. However, analysis of RNA sequencing data from The Cancer Genome Atlas (TCGA) shown decreased METTL14 (Additional file 3: Figure S1A) but elevated METTL3 levels (Additional file 3: Figure S1B) in human CRC tissue relative to normal tissue. Due to the inconsistency of METTL3 analysis results, we chose METTL14 for further research in this study. Tissue microarray containing all 37 pairs of cancerous and matched normal tissue was analyzed by immunohistochemical (IHC) staining (Fig. 1c). Consistently, high METTL14 expression was observed in 67.6% (25/37) of normal tissues. Meanwhile, in paired CRC tissue, only 32.4% (12/37) cases showed high METTL14 signal (*p* = 0.002). Besides, loss of METTL14 was also observed in multiple human CRC cell lines (Fig. 1d). With above evidence, we concluded that METTL14 was frequently down-regulated in human CRC and might be implicated in pathogenesis and progression of CRC.

### Loss of METTL14 predicted unfavorable prognosis of CRC patients

Next, we sought to explore the clinical significance of METTL14 by performing correlation analysis of METTL14 levels with clinicopathological features of enrolled CRC patients. As shown, low expression of METTL14 was positively correlated with larger tumor size, lymphatic invasion, remote metastasis and more advanced TNM stage (Table 1), indicating METTL14 might play an important role in modulating proliferation and invasion of CRC. Moreover, Kaplan-Meier analysis revealed that patients with low METTL14 expression exhibited unfavorable recurrence free survival (RFS, Fig. 1e). Moreover, we established an independent validation cohort by extracting RNA-sequencing and clinical data of CRC patients from GSE14333 dataset. Consistent with our results, decreased METTL14 expression also implicated worse RFS (Fig. 1f). In addition, multivariate analysis showed that METTL14 was an independent risk factor for RFS of CRC patients from our cohort (Table.2) and GEO dataset (Fig. 1g). Time-dependent ROC analysis showed that METTL14 had stronger predictive ability than TNM stage, especially in predicting 24-month RFS (Fig. 1h, Additional file 4: Figure S2A). Furthermore, TCGA data also showed that METTL14 was positively correlated with OS (Additional file 4: Figure S2B) and represented as an independent risk factor (Additional file 4: Figure S2C). These results suggested METTL14 is a reliable prognostic marker of CRC patients.

**Table 1:** Relationship between METTL14 and clinicopathologic factors of CRC patients

Variables	Case (*N* = 37)	METTL14 level		*p* value
		High(*n* = 12)	low(*n* = 25)	
Gender				0.85
Male	27	9	18	
Female	10	3	7	
Age				0.40
≤ 60	16	4	12	
> 60	21	8	13	
Location				0.638
Ascending colon	9	3	6	
Transverse colon	11	2	9	
Descending colon	2	1	1	
Sigmoid colon and Rectum	15	6	9	
Tumor size				0.003
≤ 5 cm	15	9	6	
> 5 cm	22	3	19	
Local invasion				0.127
T1 + T2	15	7	8	
T3 + T4	22	5	17	
Lymphatic invasion				0.001
N0	10	8	2	
N1 + N2	27	4	23	
Remote metastasis				0.042
M0	30	12	18	
M1	7	0	7	
TNM stage				0.001
I + II	10	8	2	
III+ IV	27	4	23

**Table 2:** Univariate and multivariate analysis of clinicopathologic factors for RFS of CRC patients in our cohort

	Univariate analysis			Multivariate analysis		
Variables	Hazard ratio	*P* value	95%CI	HR	*P* value	95%CI
Age (≤60 vs > 60)	1.71	0.309	0.61–4.76			
Gender	0.875	0.760	0.37–2.06			
Location						
Ascending colon	1.40	0.574	0.43–4.48			
Transverse colon	2.38	0.156	0.72–7.90			
Descending colon	0.82	0.856	0.90–7.10			
Sigmoid colon and Rectum	1.00	0.490	..			
Tumor size (≤5 cm vs > 5 cm)	2.33	0.128	0.78–6.91			
Stage (I + II vs III+ IV)	2.16	0.176	0.71–6.55			
Local invasion (T1 + T2 vs T3 + T4)	0.525	0.140	0.22–1.23			
Lymphatic invasion (N0 vs N1 + N2)	2.16	0.176	0.71–6.55			
Remote metastasis (M0 vs M1)	6.35	0.001	2.13–18.91	7.02	0.008	1.65–29.87
METTL14 level (low vs high)	6.26	0.014	1.44–27.18	21.02	0.011	2.04–216.78

### Knockdown of METTL14 promoted proliferation and invasion of CRC

**Fig. 2:**
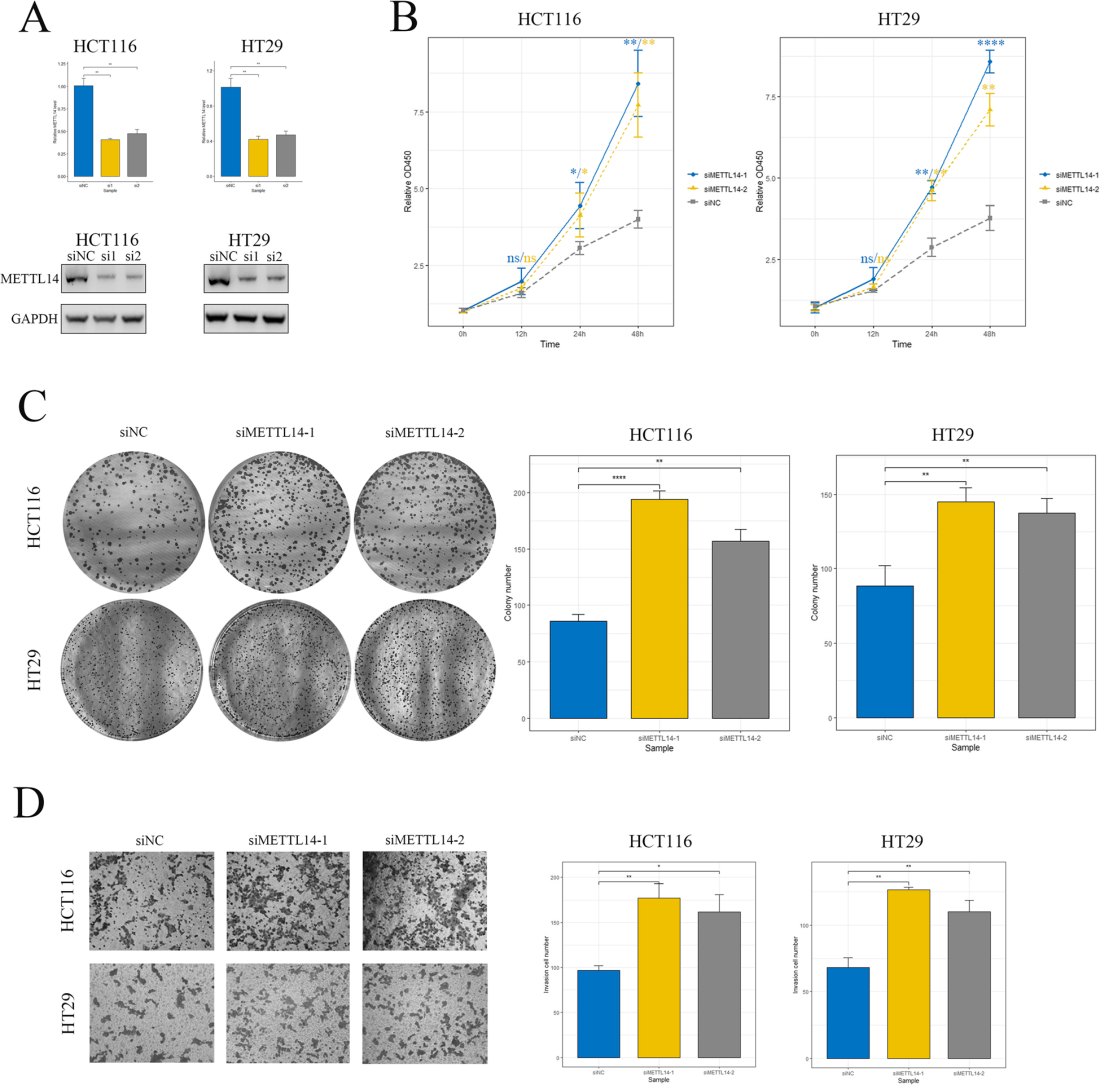
Knockdown of METTL14 promoted proliferation and invasion of CRC cells in vitro***.*a** Knockdown of METTL14 were confirmed by real-time PCR (upper panel) and western blot (lower panel) in HCT116 and HT29 cells. si1, siMETTL14–1. si2, siMETTL14–2. **, *p* < 0.01. **b** Cell count assay was performed to measure the proliferation of CRC cells transfected with siMETTL14 compared with those transfected with siNC. OD450 values were compared at indicated time points. *, *p* < 0.05, **, *p* < 0.01. ****, *p* < 0.0001. **c** Representative images and quantification of colony formation assay of HCT116 and HT29 cells transfected with siMETTL14 or siNC. Magnification, 200×. **, *p* < 0.01, ****, *p* < 0.0001. **d** Transwell invasion assay was performed to determine the effects of METTL14 on invasive ability of CRC cells. Magnification, 200×. *, *p* < 0.05, **, *p* < 0.01. All data were representative of three independent experiments and shown as mean ± SD. *P* values were determined by Student’s *t*-test

**Fig. 3:**
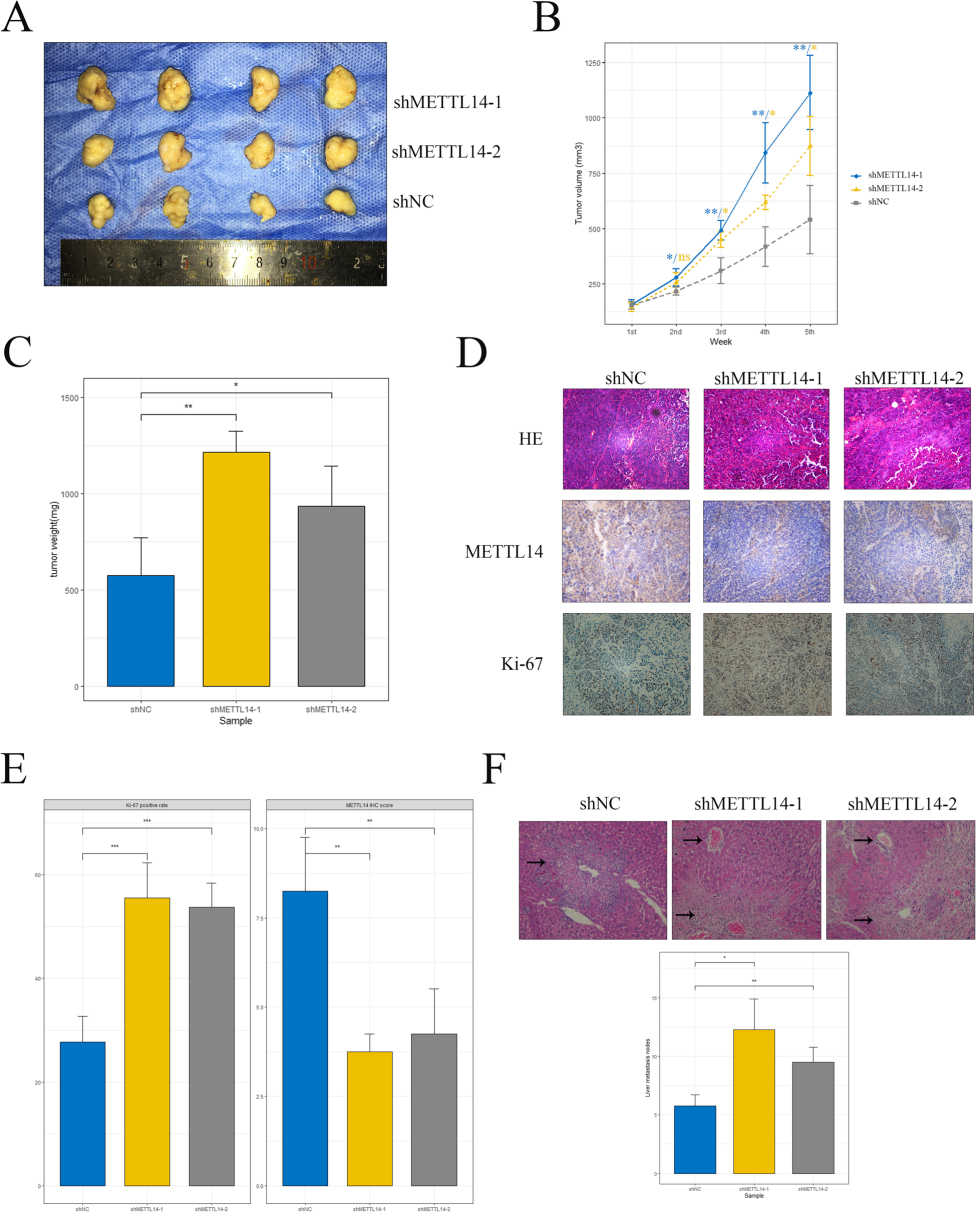
Knockdown of METTL14 promoted proliferation and invasion of CRC in vivo. **a** Xenograft tumors formed by HCT116-shMETTL14 or -shNC cells in nude mice. **b**-**c** Quantitative analysis of tumor volume (**b**) and tumor weight (**c**) of xenografts. Tumor volume was compared at indicated time points and tumor weight was measured at the end point. *, *p* < 0.05, **, *p* < 0.01. **d** Xenograft tumor sections stained with hematoxylin and eosin (HE), METTL14 and Ki-67 were examined by immunohistochemistry. Magnification, 200×. **e** Analysis of METTL14 IHC score and Ki-67 positive rate in xenograft tumor tissue from different groups. **, *p* < 0.01. **f** Analysis of metastatic liver nodules in different group of mice. Liver tissue of mice were counterstained with HE and typical metastatic lesions were indicated with black arrow. **, *p* < 0.01. *P* values were determined by Student’s *t*-test

Given the fact that METTL14 is down-regulated in tumor tissue, we speculated that METTL14 may act as a tumor suppressor in CRC. To define the functional roles of METTL14 in CRC, we established METTL14-knockdown cell model in HCT116 and HT29 cells with two independent siRNAs (Fig. 2a, Additional file 4: Figure S2D). The alteration of proliferative ability of CRC cells was then evaluated by cell count assay and colony formation assay, respectively. As expected, depletion of METTL14 markedly enhanced proliferative ability of CRC cells, reflected by expediated growth rate (Fig. 2b) and increase of colony number (Fig. 2c) of siMETTL14 cells. In addition, transwell invasion assay showed that knockdown of METTL14 dramatically promoted invasive ability of CRC cells (Fig. 2d). Moreover, two shRNAs targeting METTL14 (Additional file 4: Figure S2E) were designed and transfected into CRC cell lines to further validate the alterations of phenotypes of CRC cells. Consistently with above results, shMETTL14 cells demonstrated enhanced growing (Additional file 5: Figure S3A, S3B) and invasive (Additional file 5: Figure S3C) ability compared to control cells in cell count assay, colony formation assay and transwell invasion assay. To test our in vitro findings, we established shMETTL14 cell models and injected HCT116-shMETTL14 and control cells subcutaneously in the flank of nude mice to evaluate the effect of METTL14 on CRC tumorigenicity (Fig. 3a). Consistent with our in vitro observations, knockdown of METTL14 resulted in significant increase of the tumor volume (Fig. 3b) and tumor weight (Fig. 3c). Besides, significant higher ki-67 rate was observed in the xenograft tumors formed by shMETTL14 cells by IHC staining (Fig. 3d and e). Furthermore, we generated a liver metastasis model by injecting tumor cells into the spleen of nude mice. As expected, more metastatic nodules were found in shMETTL14 group compared to their control counterparts (Fig. 3f). Collectively, these results suggested knockdown of METTL14 promoted proliferation and invasion of CRC in vitro and in vivo.

### METTL14 inhibited CRC growth and metastasis by targeting lncRNA XIST

**Fig. 4:**
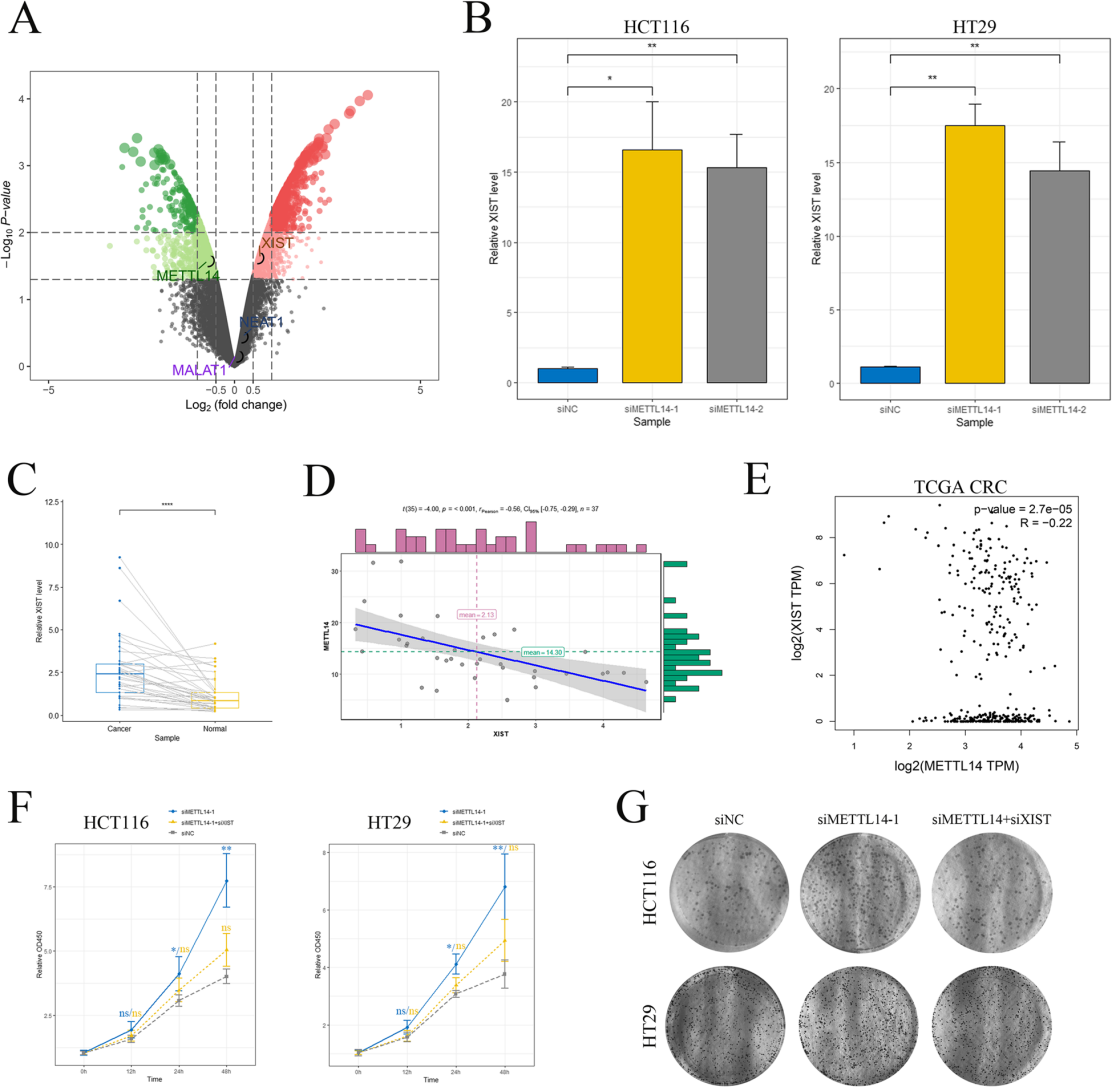
METTL14 suppressed the growth and invasion of CRC by targeting lncRNA XIST. **a** Volcano plot showed DEGs between shMETTL14 and control cells. METTL14 and interested genes which were reported to possess multiple m6A residues were indicated with black circles. **b** The levels of *XIST* were examined by real-time PCR in HCT116 and HT29 cells transfected with siNC or siMETTL14, respectively. **, *p* < 0.01. **c** Real-time PCR showing expression levels of *XIST* in CRC tissue and paired normal tissue. ****, *p* < 0.0001. **d** and **e** Negative correlation of *METTL14* and *XIST* expression in CRC tissue from our center (**d**) or from TCGA public dataset (**e**). R and *p* values were as indicated. **f** and **g** Cell count assay and transwell assay showed the effects of METTL14 and *XIST* on the proliferative and invasive ability of CRC cells. Inhibition of *XIST* suppressed the increased cell growth (**f**) and invasive ability (**g**) resulted from knockdown of *METTL14*. *, *p* < 0.05. **, *p* < 0.01. All data were representative of three independent experiments. Means ± SD were shown. Statistical analysis was conducted using Student’s *t*-test. Correlation of *METTL14* and *XIST* was evaluated by Pearson’s correlation analysis

We next sought to investigate the mechanism by which METTL14 suppressed malignant phenotype of CRC cells. As a key m6A methyltransferase, METTL14 induces installation of methyl group on m6A residues of target RNAs and activates downstream signaling. Moreover, recent studies demonstrated that pri-miRNAs could be marked by m6A-methylation for subsequent splicing and maturing [22]. Besides, m6A process was also proved to participate in modulating process of lncRNAs [23]. These reports not only highlight the important role of m6A modification in RNA processing, but also remind us that m6A-methylation can affect cancer biology by regulating non-coding RNAs (ncRNAs). We then conducted gene set enrichment analysis (GSEA) to explore the potential downstream pathways of METTL14. As shown, METTL14 was positively correlated with ncRNA metabolism (Additional file 6: Figure S4A), ncRNA processing (Additional file 6: Figure S4B) and RNA degradation (Additional file 6: Figure S4C). In contrast, negative correlation was seen between METTL14 and several signaling pathways namely growth factor binding, collagen trimer and collagen binding (Additional file 6: Figure S4D-4F). Therefore, METTL14 may exert anti-tumor activity via targeting and down-regulating oncogenes or oncogenic lncRNAs in these pathways. We then performed RNA-seq to interrogate the expression changes in HCT116-shMETTL14 cells relative to the control cells. The fold change of gene expression was calculated and genes with log2|FC| *>* 0.5 were considered as differentially expressed. Among thousands of potential targets, we noticed lncRNA *XIST* was significantly upregulated upon METTL14 knockdown (Fig. 4a, Additional file 7: Figure S5A). *XIST* is a potent oncogenic lncRNA expediting growth and metastasis of CRC cells [19]. Importantly, analysis of transcriptome m6A mapping data revealed that at least 78 m6A residues are located across *XIST* sequence (Additional file 7: Figure S5B and Additional file 1: Table S1) [20]. As typical mRNA only contains 3–5 m6A sites, the high abundance of m6A sites of *XIST* implied an incredibly important role of m6A in regulating of *XIST* function. Thus, we assumed that METTL14 inhibited CRC growth and metastasis by down-regulating lncRNA *XIST*. To verify this hypothesis, expression levels of *XIST* was first detected in METTL14 knockdown and control cells. As expected, METTL14 depletion led to distinct elevation of *XIST* in two CRC cell lines (Fig. 4b). Next, we quantified *XIST* levels in cancer and normal specimens. Consistent with reported results, *XIST* exhibited higher levels in cancer compared to normal tissues (Fig. 4c), which suggested a tumor-driving effect of *XIST* in CRC. Moreover, a negative correlation was observed between expression levels of METTL14 and *XIST* in CRC samples from our cohort (Fig. 4d). Besides, TCGA RNA-seq data of CRC also showed that *XIST* upregulation often happened in cases with downregulation of METTL14 (Fig. 4e). Additionally, enhanced cell growth (Fig. 4f, g and Additional file 7: Figure S5C) and invasion (Additional file 7: Figure S5D) induced by METTL14 knockdown could be attenuated by *XIST* inhibition. Notably, these results were also tested in shMETTL14 cells to confirm that knockdown of *XIST* diminished the growth (Additional file 8: Figure S6A, S6B) and invasion (Additional file 8: Figure S6C) of shMETTL14 cells. Taken together, these results indicated that increased CRC growth and invasion resulted from METTL14 depletion was, at least partly, due to elevation of lncRNA *XIST*.

### METTL14 down-regulated XIST through a m6A-YTHDF2 dependent pathway

**Fig. 5:**
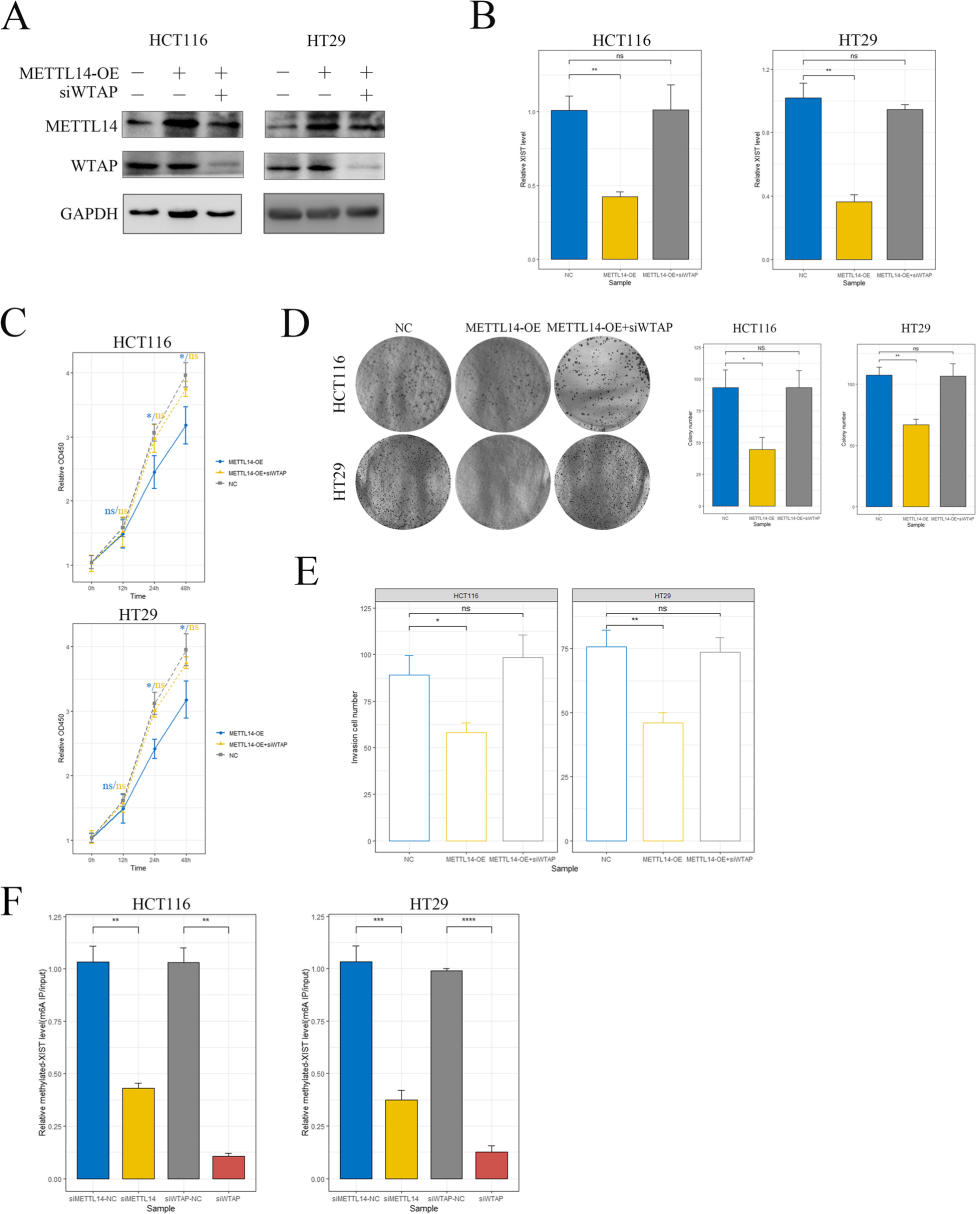
METTL14 down-regulated XIST through m6A methylation activity. **a** Two CRC cell lines were transfected with METTL14-overexpression (METTL14-OE) plasmids and siWTAP as indicated. Protein band intensity of METTL14 and WTAP were quantified by western blot assay and shown as fold change. Statistical histograms were not shown. **b** The alteration of *XIST* expression in METTL14-OE and METTL14-OE + siWTAP cells was analyzed by real-time PCR. ns, not significant, **, *p* < 0.01. **c**-**d** The effects of WTAP and METTL14 on proliferative ability of CRC cells were measured by cell count assay (**c**) and colony formation assay (**d**). ns, not significant, *, *p* < 0.05, **, *p* < 0.01. **e** Quantitative analysis of transwell invasion assay showing the effects of METTL14 and WTAP on invasive ability of CRC cells. ns, not significant, *, *p* < 0.05, **, *p* < 0.01. **f** Quantitative analysis of m6A-methylated *XIST* in control, siMETTL14–1 and siWTAP cells. Methylated *XIST* was immunoprecipitated with m6A antibody and then measured by real-time PCR. Equal amount of total RNA was used as input. **, *p* < 0.01, ***, *p* < 0.001, ****, *p* < 0.0001. All data were representative of three independent experiments. Means ± SD were shown. P values were determined by Student’s *t*-test

We next plan to clarify whether the function of METTL14 down-regulating *XIST* directly depends on its m6A catalytic activity. METTL14 was ectopically over-expressed in HCT116 and HT29 cells (Fig. 5a, Additional file 9: Figure S7A), the potential alterations of *XIST* expression level and cell phenotypes were then assessed. As shown, overexpression of METTL14 resulted in remarkable decrease in *XIST* expression level (Fig. 5b), cell growth (Fig. 5c, d) and invasion (Fig. 5e and Additional file 9: Figure S7B) of CRC cells, which were consistent with the above results and confirmed the suppressive effects of METTL14 on CRC. Recently, METTL3 and METTL14 are identified as interactors with Wilms tumor-associated protein (WTAP). WTAP acts as a scaffold and binds the methyltransferase to form a complex which mediates m6A methylation on RNAs. The loss of WTAP significantly blocks m6A modification process. Thus, we performed WTAP knockdown with siRNA in METTL14-overexpressed (METTL14-OE) cells (Fig. 5a, Additional file 9: Figure S7A) to inhibited the cellular m6A process and analyzed the effect of this act. As shown, the decreased *XIST* level induced by METTL14 overexpression could be attenuated by WTAP depletion (Fig. 5b). In line with this, WTAP knockdown significantly rescued the impaired growth (Fig. 5c and d) and invasion (Fig. 5e and Additional file 9: Figure S7B) of METTL14-OE cells. These results suggested that the downregulation of *XIST* induced by METTL14 was dependent on the formation of m6A complex and subsequent m6A methylation process. Moreover, m6A-methylated *XIST* was immunoprecipitated with m6A antibody in siMETTL14 and siWTAP cell lysates and then quantified with real-time PCR. We found that knockdown of METTL14, or WTAP led to significant reduced levels of methylated *XIST* (Fig. 5f). These results directly showed that METTL14 promotes m6A-methylation of *XIST*, resulting in downregulation of this lncRNA and suppression of CRC proliferation and invasion. Intriguingly, we noticed that knockdown of WTAP almost totally abolished the m6A-methylation of *XIST* while METTL14 depletion significantly but only partly decreased m6A levels (Fig. 5f), which suggested that there might be other mechanisms involved.

**Fig. 6:**
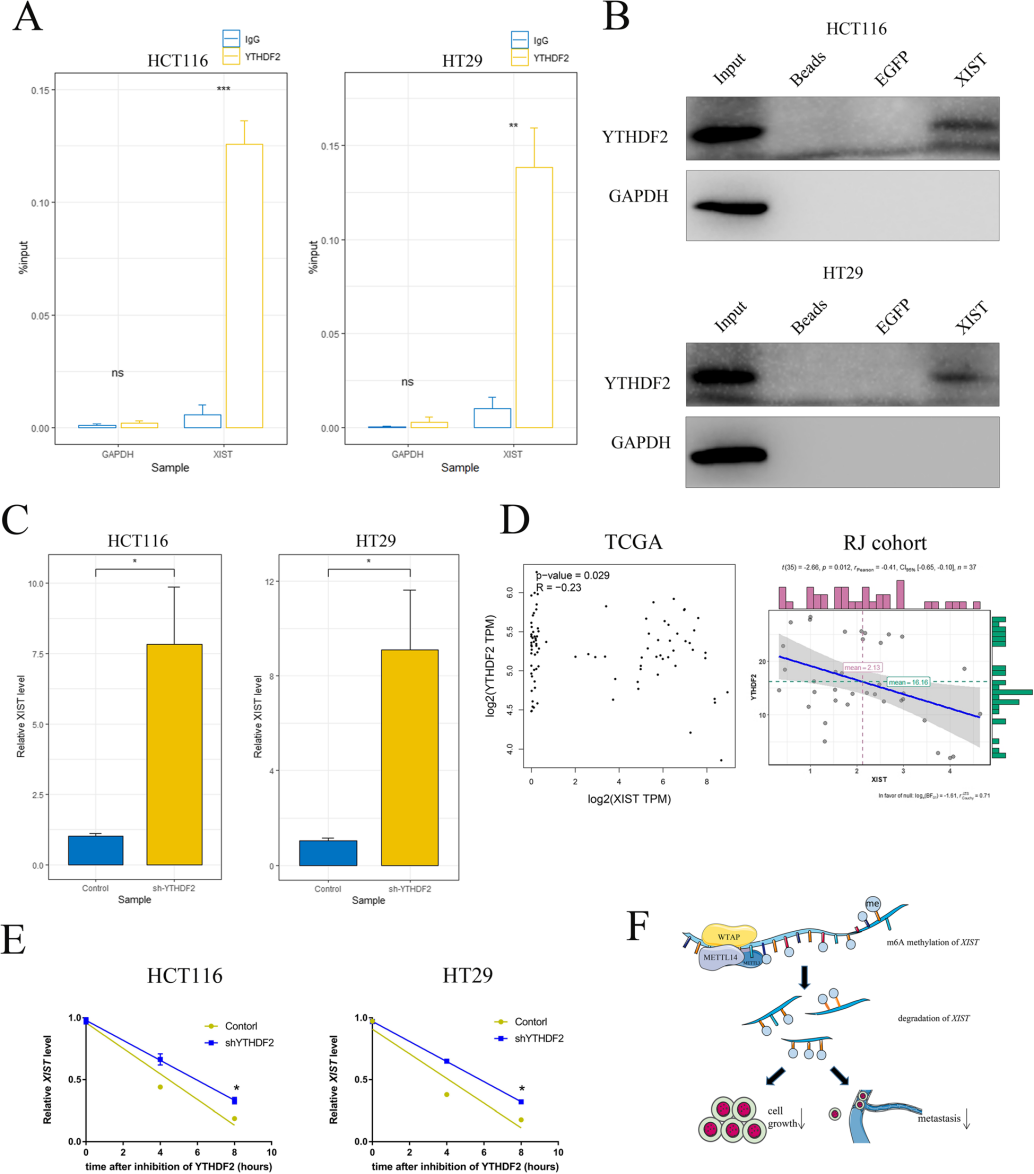
YTHDF2 mediated the recognition and degradation of m6A-methylated XIST. **a** RIP assay confirmed the association between *XIST* and m6A reader protein YTHDF2 in CRC cells. GAPDH mRNA was used as a non-target control. ns, not significant, **, *p* < 0.01, ***, *p* < 0.001. **b** RNA pull-down assay confirmed *XIST* was specifically recognized by YTHDF2. EGFP RNA was used as RNA control. GAPDH was used as protein control. **c** Real-time PCR showed the level of *XIST* in control and shYTHDF2 CRC cells. *, *p* < 0.05. **d** Negative correlation of *METTL14* and *XIST* expression in CRC tissue from our center (right panel) or from TCGA public dataset (left panel). R and p values were as indicated. **e** The expression levels of *XIST* in shYTHDF2 and control cells were quantified by real-time PCR at indicated time points after actinomycin D treatment and the decay rate of *XIST* was evaluated with a linear regression model. *, *p* < 0.05. **f** Summary of the mechanism by which METTL14 regulates the malignant phenotype of CRC. Data were presented as the mean ± SD of at least three independent experiments. P values were determined by Student’s *t*-test

m6A methylation is a marking procedure which needs to be recognized by m6A “reader” proteins for further disposition of target RNAs [24]. We next investigated the reader protein which recognized the m6A-methylation of *XIST* in order to mediate its downregulation. Analysis of previously reported iCLIP-sequencing data identified direct binding between *XIST* and the m6A readers YT521-B homology (YTH) domain family [20] which comprise three members of the YTHDF proteins (YTHDF1, YTHDF2, and YTHDF3), YTHDC1 and YTHDC2. To determine the binding between *XIST* and the potential reader proteins in CRC cells, we immunoprecipitated YTH family proteins with proper antibodies from the cell lysates and then measured the amount of bound *XIST* by quantitative PCR. As shown, YTHDF2 immunoprecipitants contained significantly higher level of *XIST* than control group (Fig. 6a and Additional file 10: Figure S8A). Consistently, RNA pull-down assay showed that YTHDF2, but not other readers, was significantly enriched by biotin-labeled *XIST* (Fig. 6b and Additional file 10: Figure S8B). Given that YTHDF2 was reported to facilitate m6A-dependent RNA degradation under normal and stress conditions [25], we hence speculated that YTHDF2-dependent m6A-RNA decay played an important role in the downregulation of *XIST*. As expected, knockdown of YTHDF2 resulted in remarkable augment of *XIST* expression in HCT116 and HT29 cells (Fig. 6c), which validated our hypothesis. Moreover, expression levels of YTHDF2 and *XIST* was negatively correlated with each other in TCGA dataset and our cohort (Fig. 6d). In addition, the decay rate of *XIST* was significantly slower in shYTHDF2 CRC cells, which directly showed YTHDF2 mediated the degradation of lncRNA *XIST* (Fig. 6e and Additional file 10: Figure S8C). Taken together, these findings suggested that METTL14-induced m6A process suppressed *XIST* expression through YTHDF2-dependent RNA degradation.

## Discussion

N6-methyladenosine (m6A) of RNA has been proposed as a new layer of epigenetic regulation. This biochemical process is proved to play important roles in regulation of cell growth, differentiation, and self-renewal through controlling RNA splicing, translation and stability [23, 26, 27]. Previous studies identified m6A as a predominant modification of mRNAs [7, 23]. Strikingly, recent studies discovered that m6A was also present in the modification of ncRNAs. Among them, lncRNA *XIST* is highly methylated at special sites and m6A modifications were indispensable for *XIST*-mediated gene silencing. Accordingly, knockdown of methyltransferase like 3 (METTL3), or other accessory components including WTAP, RMB15 and RMB15B, significantly impaired *XIST*-mediated gene silencing [20].

Given the fact that lncRNA *XIST* promotes growth and invasion of colorectal cancer [19] and possesses abundant m6A sites (Additional file 7: Figure S5B and Additional file 1: Table S1) [20], it is reasonable for us to speculate that m6A may play an important role in regulating the biological behavior of CRC cells through inducing methylation of *XIST*. In the present study, we observed a decreased tendency of METTL14 from normal colon tissue to CRC (Fig. 1), suggesting the potential role of m6A in CRC tumorigenesis. Moreover, we demonstrated that METTL14 downregulation resulted in enhanced tumor growth and invasion of CRC both in vitro and in vivo (Fig. 2 and Fig. 3). In addition, METTL14 could serve as a prognostic factor for CRC patients (Fig. 1). Most importantly, we identified that lncRNA *XIST* was a direct downstream target of METTL14-mediated m6A modification (Fig. 4 and Fig. 5), unveiling the mechanisms by which METTL14 manipulated proliferative and invasive ability of CRC cells (Fig. 6f).

m6A process refers to a reversible chemical modification on RNA which is driven by a complex containing two catalytic components, METTL3 and METTL14. They form a heterodimer and catalyze the covalent transfer of methyl group to adenine with the assistance of WTAP, accomplishing the m6A installation process [28, 29]. Previous studies suggested that dysregulation of these two enzymes contributed to human carcinogenesis. METTL3 was conspicuously upregulated in hepatocellular carcinoma (HCC) and consequently promoted proliferation and metastasis of HCC via silencing of tumor suppressor SOCS2 [11]. METTL3 was also elevated in other solid tumors such as breast cancer [30], pancreatic cancer [31] and promotes growth, invasion and drug resistance of cancer cells. In contrast, METTL14 was down-regulated in HCC and resulted in decreased m6A level and enhanced the metastatic capacity of HCC cells [12]. In agreement with previous report, the present study also identified METTL14 as a tumor suppressor gene in CRC through down-regulating oncogenic lncRNA *XIST*, suggesting that m6A also represent a distinct form of epigenetic dysregulation in colorectal cancer. Moreover, although TCGA data showed that abnormal expression of METTL3 may exist in CRC, our knowledge about the link between METTL3 and CRC is still limited. A recent study reported that METTL3 could promote self-renewal of CRC cells through inducing m6A and subsequent upregulation of *SOX2* [32]. Similarly, METTL3 and METTL14 also showed opposite effects in the regulation of HCC as above mentioned [11, 12]. However, as METTL3 and METTL14 have to form a heterodimer to induce m6A methylation, it seemed to be contradictory for two components of a functional complex demonstrated definitely opposite effects on progression of cancer cells. We speculated that METTL3 and METTL14 might prefer different targets and lead to diverse downstream pathways. Moreover, the methyltransferase activating and determining the cell fate under certain conditions may have tissue and cellular specificity.

Whereas methyltransferase serve as the ‘writer’ of m6A, there are demethylases, FTO and alkB homolog 5 (ALKBH5), act as the ‘eraser’ to reverse the methylation. ALKBH5 catalyzes the direct removal of m6A [10], while FTO can sequentially oxidize m6A to N6-hydroxymethyladeosine (hm6A) or N6-formyladenosine (f6A) [33], which are unstable and can be hydrolyzed to adenine. Notably, dysregulations of demethylases were also reported in cancers. FTO played an oncogenic role in acute myeloid leukemia through regulating target genes such as *ASB2* and *RARA* by reducing m6A levels in these mRNA transcripts [14]. ALKBH5 promoted cancer cell renewal and growth in breast cancer by removing m6A from NANOG mRNA, which in turn enhanced NANOG mRNA stability and pluripotency of cancer cells [34].

Similar to DNA methylation, the biological function of m6A is mediated through the selective recognition of m6A sites by the “reader” proteins [6, 24]. Among which, YTHDF2 is the first identified and the most extensively studied m6A reader. YTHDF2 can bind to m6A residues located in the untranslated region through C-terminal YTD domain and render the targeted mRNA to processing bodies for subsequent degradation [35, 36]. Unlike YTHDF2, YTHDF1 promotes mRNA translation efficiency by interacting with EIF3 to promote rate-limiting step of translation for m6A-modified mRNAs [37]. YTHDC1 function as a regulator of RNA alternative splicing by recruiting serine and arginine-rich splicing factors (SRSFs) to its mRNA-binding regions near m6A sites [38]. Furthermore, heterogeneous nuclear ribonucleoprotein (hnRNP) could also serve as m6A reader to influence mRNA localization, alternative splicing and microRNA processing [39, 40]. Previous study reported that YTHDC1 recognized the METTL3-induced m6A methylation of *XIST* to mediate the transcriptional silencing of genes on the X chromosome [20]. However, here we found that m6A-methylated *XIST* was selectively recognized by YTHDF2 instead of YTHDC1, which was different from the results of previous study. We assumed that METTL14 might target different m6A sites of *XIST* comparing with METTL3, which led to diverse interactions with reader proteins and distinct downstream reactions. This question needs to be further clarified by single-nucleotide m6A detection methods.

Recent transcriptome-wide m6A profiling studies identified non-coding RNAs as both targets and regulators of m6A methyltransferases [23, 41, 42]. Subsequent studies established a model that METTL3 and METTL14 marked primary microRNAs for processing and maturation [12, 22]. Additionally, further investigations found that m6A-methylation was also presented in lncRNAs with m6A sites, such as *XIST*, *NEAT1*, [20], *MALAT1* [40], and lincRNA1281 [43] and was responsible for the stability, structure, and function of these lncRNAs. Consistently, our proposition that m6A might target lncRNAs to affect cancer progression was validated in CRC cells. However, the above results indicated a complicated regulating network of m6A in different models of diseases. It is currently unclear what determines the target specificity and cellular heterogeneity of m6A modification in cancer cells. In addition, we wonder whether dysregulation of FTO or ALKBH5 exists in CRC and contributes to tumor formation or progression. Moreover, compared to recent advances in this aspect, the detailed m6A sites on *XIST* by which METTL14 targeted and regulated CRC cells was not precisely located in the present study. In addition, as most lncRNAs including *XIST* were produced in cell nuclei, the mechanisms mediating the export of *XIST* to cytoplasm for degradation was still not clear. Future investigations are necessary to answer these questions.

## Conclusion

Collectively, we identified a ‘METTL14-YTHDF2-lncRNA’ regulating axis in CRC cells, which presented a systemic vision of the function and mechanism of m6A in CRC and revealed a novel dimension of cancer biology. Besides, by highlighting the crucial role and prognostic value of METTL14, our discovery may pave the way for developing new therapeutic strategies against CRC.

## Supplementary information

**Additional file 1: Table S1.** m6A sites along *XIST* predicted by Me-DB online tools.

**Additional file 2: Table S2.** plasmids or siRNAs.

**Additional file 3: Figure S1.** (A-B) METTL14 was significantly down-regulated (A) while METTL3 was up-regulated (B) in CRC. RNA sequencing data was from TCGA database. *P* values were as shown.

**Additional file 4: Figure S2.** (A) Comparison of predictive ability of METTL14 and TNM stage of RFS of CRC patients at 24 months. AUC values were as shown. (B) CRC patients from TCGA colon adenocarcinoma (COAD) and rectal adenocarcinoma (READ) dataset were classified into METTL14-high and -low group and difference between two groups was compared with Kaplan-Meier analysis. (C) Multivariate analysis of TCGA data showed that METTL14 was an independent risk factor for OS of CRC patients. (D) Quantitative analysis of western blots of Fig.2a. (E) Knockdown of METTL14 was performed with two shRNAs and was confirmed by western blots.

**Additional file 5: Figure S3.** The proliferative and invasive ability of two shMETTL14 cells were tested by cell count assay (A), colony formation assay (B) and transwell assay (C). ns, not significant, *, *p* < 0.05, ****, *p* < 0.0001.

**Additional file 6: Figure S4.** (A-C) METTL14 was positively correlated with regulation of non-coding RNA (ncRNA) metabolism (A), ncRNA processing (B), and RNA degradation (C). (D-F) METTL14 was negatively associated with growth factor binding (D), collagen trimer (E) and collagen binding (F). P values and normalized enrichment scores (NES) were as indicated.

**Additional file 7: Figure S5.** (A) Heatmap showed the DEGs between shMETTL14 and control cells. (B) Schematic diagram of m6A methylation sites of lncRNA *XIST.* Analysis was carried out by online tool MeT-DB (http://compgenomics.utsa.edu/methylation/). Potential m6A sites were shown as vertical bars. Three independent algorithms were applied. Details were shown in Additional file 1: Table S1. (C) Statistical histograms of Fig.4g. (D) Inhibition of *XIST* could attenuated the enhanced invasion of CRC cells resulted from METTL14 knockdown.

**Additional file 8: Figure S6.** Inhibition of *XIST* was performed in shMETTL14 cells and the proliferative and invasive ability were then tested by cell count assay (A), colony formation assay (B), transwell invasion assay (C), respectively. The results showed that inhibition of *XIST* attenuated the increased cell growth and invasion of shMETTL14 cells.

**Additional file 9: Figure S7.** (A) Statistical analysis of protein bands from Fig.5a. (B) Representative images of transwell invasion assay of CRC cells with different expression status of METTL14 and WTAP.

**Additional file 10: Figure S8.** RIP (A) and RNA-pulldown (B) assay detected negligible binding between *XIST* and YTHDF1, YTHDF3, YTHDC1 or YTHDC2. ns, not significant. (C) Knockdown of YTHDF2 was confirmed with western bolts.

## Data Availability

All data generated during this study are included either in article or in the additional files. RNA-seq data will be uploaded to GEO database in the future.

## Abbreviations

CRC: colorectal cancer
GSEA: Gene set enrichment analysis
lncRNA: long non-coding RNA
m6A: N6-methyladenosine
MeRIP: Methylated RNA immunoprecipitation
METTL14: methyltransferase-like14
RFS: recurrence free survival
WTAP: Wilms tumor-associated protein
XIST: X inactivate-specific transcript
YTHDF2: YT521-B homology domain family 2
