# Supplementary material for: METTL14 suppresses proliferation and metastasis of colorectal cancer by down-regulating oncogenic long non-coding RNA XIST
Source: Mol Cancer. 2020 Feb 28;19:46. doi: 10.1186/s12943-020-1146-4 (PMC7047419; doi:10.1186/s12943-020-1146-4)
Supplement: Supplementary file 1 — Additional file 1: Table S1. m6A sites along XIST predicted by Me-DB online tools. [file 12943_2020_1146_MOESM1_ESM.docx]

Table.S1 m6A sites along *XIST* predicted by Me-DB online tools.

| methylationType | source | chromStart | chromEnd | Enrichment Score |
| --- | --- | --- | --- | --- |
| m6A | p007_human_HeLa1_KO_M14 | 55270342 | 55270402 | 1.49 |
| m6A | p007_human_HeLa1_ctrl | 55278725 | 55278874 | 1.84 |
| m6A | p007_human_HeLa1_KO_WTAP | 55220268 | 55221764 | 1.91 |
| m6A | p007_human_HeLa1_KO_WTAP | 55231468 | 55236249 | 2.09 |
| m6A | p002_human_HepG2_HGF | 55221793 | 55223531 | 2.1 |
| m6A | p004_human_u2os_daa | 55538783 | 55540726 | 2.2 |
| m6A | p004_human_u2os_daa | 55750282 | 55750462 | 2.2 |
| m6A | p007_human_HeLa1_KO_M14 | 55278725 | 55278874 | 2.25 |
| m6A | p007_human_HeLa1_KO_WTAP | 55233059 | 55233177 | 2.26 |
| m6A | p007_human_HeLa1_KO_WTAP | 55270222 | 55270462 | 2.53 |
| m6A | p007_human_HeLa1_KO_WTAP | 55539829 | 55540069 | 2.64 |
| m6A | p007_human_HeLa1_ctrl | 55499020 | 55499229 | 2.73 |
| m6A | p007_human_HeLa1_KO_WTAP | 55539470 | 55539740 | 2.73 |
| m6A | p007_human_HeLa2_ctrl | 55270282 | 55270462 | 2.73 |
| m6A | p004_human_u2os_ctrl | 55538783 | 55540726 | 2.77 |
| m6A | p007_human_HeLa1_KO_WTAP | 55240683 | 55240804 | 2.79 |
| m6A | p002_human_HepG2_HS | 55266435 | 55266526 | 2.84 |
| m6A | p004_human_u2os_daa | 55756635 | 55759024 | 2.85 |
| m6A | p007_human_HeLa2_KO_M3 | 55498930 | 55499229 | 2.85 |
| m6A | p007_human_HeLa1_KO_M14 | 55539411 | 55539710 | 2.98 |
| m6A | p007_human_HeLa1_ctrl | 55240683 | 55240774 | 3.11 |
| m6A | p004_human_u2os_ctrl | 55273148 | 55273209 | 3.16 |
| m6A | p007_human_HeLa1_KO_WTAP | 55538843 | 55539173 | 3.22 |
| m6A | p007_human_HeLa1_KO_WTAP | 55540337 | 55560082 | 3.23 |
| m6A | p001_human_S3_NEB_HiSeq | 55273835 | 55273985 | 3.31 |
| m6A | p004_human_u2os_ctrl | 55274014 | 55274135 | 3.4 |
| m6A | p007_human_HeLa1_KO_M14 | 55540247 | 55560022 | 3.41 |
| m6A | p004_human_u2os_daa | 55273955 | 55274135 | 3.43 |
| m6A | p001_human_S1_SYSY_GAII | 55278784 | 55279053 | 3.44 |
| m6A | p007_human_HeLa1_KO_M14 | 55539889 | 55540159 | 3.47 |
| m6A | p007_human_HeLa1_KO_M14 | 55538843 | 55539202 | 3.85 |
| m6A | p007_human_HeLa1_KO_M14 | 55270760 | 55273657 | 3.9 |
| m6A | p007_human_HeLa2_KO_M3 | 55979584 | 55979645 | 3.91 |
| m6A | p001_human_S2_SYSY_HiSeq | 55498960 | 55499229 | 4.38 |
| m6A | p007_human_HeLa2_KO_M3 | 55539351 | 55540726 | 4.43 |
| m6A | p001_human_S2_SYSY_HiSeq | 55539351 | 55540666 | 4.72 |
| m6A | p001_human_S3_NEB_HiSeq | 55498990 | 55499199 | 4.96 |
| m6A | p007_human_HeLa1_KO_M14 | 55273716 | 55274195 | 5.22 |
| m6A | p007_human_HeLa2_KO_M3 | 55278665 | 55278994 | 5.26 |
| m6A | p007_human_HeLa1_ctrl | 55539411 | 55560082 | 5.38 |
| m6A | p007_human_HeLa1_KO_WTAP | 55273716 | 55274254 | 5.4 |
| m6A | p002_human_HepG2_IFN | 55749331 | 55749481 | 5.47 |
| m6A | p001_human_S2_SYSY_HiSeq | 55979465 | 55979705 | 5.5 |
| m6A | p007_human_HeLa1_ctrl | 55538783 | 55539292 | 5.74 |
| m6A | p007_human_HeLa1_KO_WTAP | 55270730 | 55273567 | 5.76 |
| m6A | p002_human_HepG2_UV | 55498990 | 55499050 | 6.06 |
| m6A | p001_human_S1_SYSY_GAII | 55539381 | 55540696 | 6.33 |
| m6A | p002_human_HepG2_UT | 55539829 | 55540129 | 6.41 |
| m6A | p001_human_S1_SYSY_GAII | 55498930 | 55499289 | 6.54 |
| m6A | p002_human_HepG2_IFN | 55538873 | 55539083 | 6.62 |
| m6A | p001_human_S1_SYSY_GAII | 55979435 | 55980331 | 6.69 |
| m6A | p001_human_S2_SYSY_HiSeq | 55538813 | 55539202 | 6.83 |
| m6A | p007_human_HeLa1_ctrl | 55270730 | 55274314 | 6.98 |
| m6A | p002_human_HepG2_HS | 55268036 | 55270313 | 7.04 |
| m6A | p007_human_HeLa2_ctrl | 55278546 | 55279083 | 7.09 |
| m6A | p002_human_HepG2_HS | 55540187 | 55540368 | 7.15 |
| m6A | p002_human_HepG2_HS | 55540486 | 55540637 | 7.15 |
| m6A | p001_human_S3_NEB_HiSeq | 55540158 | 55540696 | 7.29 |
| m6A | p007_human_HeLa2_ctrl | 55538723 | 55559992 | 7.58 |
| m6A | p002_human_brain | 55538873 | 55539023 | 8.07 |
| m6A | p001_human_S1_SYSY_GAII | 55538813 | 55539232 | 8.75 |
| m6A | p007_human_HeLa2_KO_M3 | 55538783 | 55539232 | 9.04 |
| m6A | p002_human_HepG2_HS | 55238884 | 55241660 | 9.53 |
| m6A | p002_human_HepG2_UT | 55540277 | 55540637 | 10 |
| m6A | p007_human_HeLa2_ctrl | 55498960 | 55499319 | 10.2 |
| m6A | p002_human_HepG2_UT | 55538813 | 55539143 | 10.4 |
| m6A | p002_human_HepG2_IFN | 55863143 | 55863352 | 10.8 |
| m6A | p001_human_S3_NEB_HiSeq | 55538873 | 55539173 | 11.4 |
| m6A | p001_human_S1_SYSY_GAII | 55273028 | 55273657 | 11.8 |
| m6A | p002_human_HepG2_HGF | 55539918 | 55540039 | 12.2 |
| m6A | p001_human_S3_NEB_HiSeq | 55539411 | 55539740 | 12.3 |
| m6A | p007_human_HeLa2_KO_M3 | 55272969 | 55274493 | 13.1 |
| m6A | p007_human_HeLa2_ctrl | 55270760 | 55274523 | 14.2 |
| m6A | p007_human_HeLa2_KO_M3 | 55238307 | 55238368 | 14.2 |
| m6A | p002_human_HepG2_HS | 55233001 | 55233118 | 15.7 |
| m6A | p002_human_HepG2_HGF | 55278725 | 55279142 | 16 |
| m6A | p002_human_HepG2_UV | 55278248 | 55278339 | 16.6 |
| m6A | p002_human_HepG2_UV | 55539530 | 55539740 | 16.7 |
| m6A | p002_human_HepG2_UT | 55273148 | 55273657 | 17.2 |
| m6A | p002_human_HepG2_HS | 55539500 | 55539740 | 17.7 |
| m6A | p001_human_S2_SYSY_HiSeq | 55273775 | 55274344 | 18.2 |
| m6A | p002_human_HepG2_HS | 55220219 | 55220357 | 20.4 |
| m6A | p001_human_S1_SYSY_GAII | 55273745 | 55274374 | 21 |
| m6A | p002_human_HepG2_HS | 55229218 | 55236279 | 22.5 |
| m6A | p002_human_HepG2_UV | 55538843 | 55539202 | 24 |
| m6A | p002_human_HepG2_HS | 55249011 | 55260501 | 26.6 |
| m6A | p002_human_HepG2_HS | 55248986 | 55249194 | 26.6 |
| m6A | p001_human_S2_SYSY_HiSeq | 55273148 | 55273657 | 27.4 |
| m6A | p002_human_HepG2_HS | 55210100 | 55224485 | 30.3 |
| m6A | p002_human_HepG2_HS | 55086724 | 55087054 | 32 |
| m6A | p002_human_HepG2_HGF | 55538813 | 55539173 | 32.2 |
| m6A | p002_human_HepG2_HGF | 55540367 | 55540577 | 34.3 |
| m6A | p002_human_HepG2_UV | 55273028 | 55274195 | 35.3 |
| m6A | p002_human_HepG2_HS | 55538813 | 55539083 | 35.9 |
| m6A | p002_human_HepG2_IFN | 55273208 | 55274195 | 43.6 |
| m6A | p002_human_HepG2_UT | 55273716 | 55274314 | 66.8 |
| m6A | p002_human_HepG2_HGF | 55273148 | 55274284 | 114 |
| m6A | p002_human_HepG2_HS | 55272999 | 55274284 | 238 |
