# Supplementary material for: METTL14 suppresses proliferation and metastasis of colorectal cancer by down-regulating oncogenic long non-coding RNA XIST
Source: Mol Cancer. 2020 Feb 28;19:46. doi: 10.1186/s12943-020-1146-4 (PMC7047419; doi:10.1186/s12943-020-1146-4)
Supplement: Supplementary file 2 — Additional file 2: Table S2. plasmids or siRNAs. [file 12943_2020_1146_MOESM2_ESM.docx]

Table.S2 plasmids or siRNAs.

| Name | Oligonucleotides (5’-3’) | | |
| --- | --- | --- | --- |
|  | Forward sequence | | Reverse sequence |
| METTL14-OE | GTGCAGAAGGGACTAGGCAG | GGGCACCCACGTAATAGACC | |
|  | **target sequences** | | |
| ShMETTL14-1 | GCATTGGTGCCGTGTTAAATA | | |
| ShMETTL14-2 | GGATGAAGGAGAGACAGATGA | | |
| ShYTHDF2 | CGGTCCATTAATAACTATAAC | | |
| SiMETTL14-1 | GCTGGACTTGGGATGATATTA | | |
| SiMETTL14-2 | GAACCTGAAATTGGCAATATA | | |
| SiWTAP | GGAGGTAGTGGTTACGTAAAT | | |
| SiXIST | ACCTCTCCTTTCTCTGCCTAC | | |

For ectopic expression of METTL14, primers used for cloning METTL14 CDS were shown. For knockdown of target genes, target sequences were shown. OE, overexpression.
