# Supplementary figures and images for: METTL14 suppresses proliferation and metastasis of colorectal cancer by down-regulating oncogenic long non-coding RNA XIST
Source: Mol Cancer. 2020 Feb 28;19:46. doi: 10.1186/s12943-020-1146-4 (PMC7047419; doi:10.1186/s12943-020-1146-4)

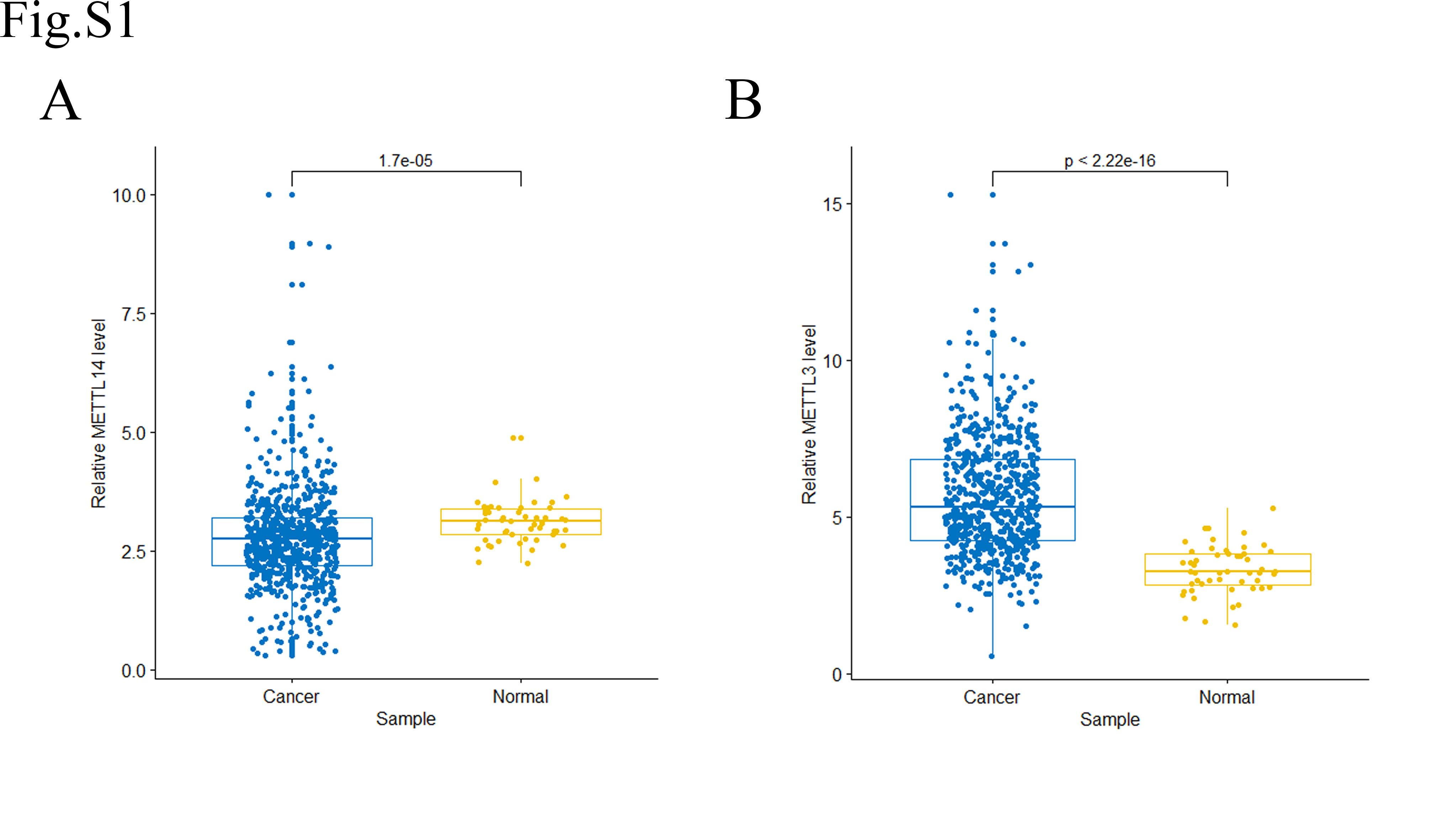

Supplement: Supplementary file 3 — Additional file 3: Figure S1. (A-B) METTL14 was significantly down-regulated (A) while METTL3 was up-regulated (B) in CRC. RNA sequencing data was from TCGA database. P values were as shown. [file 12943_2020_1146_MOESM3_ESM.jpg]

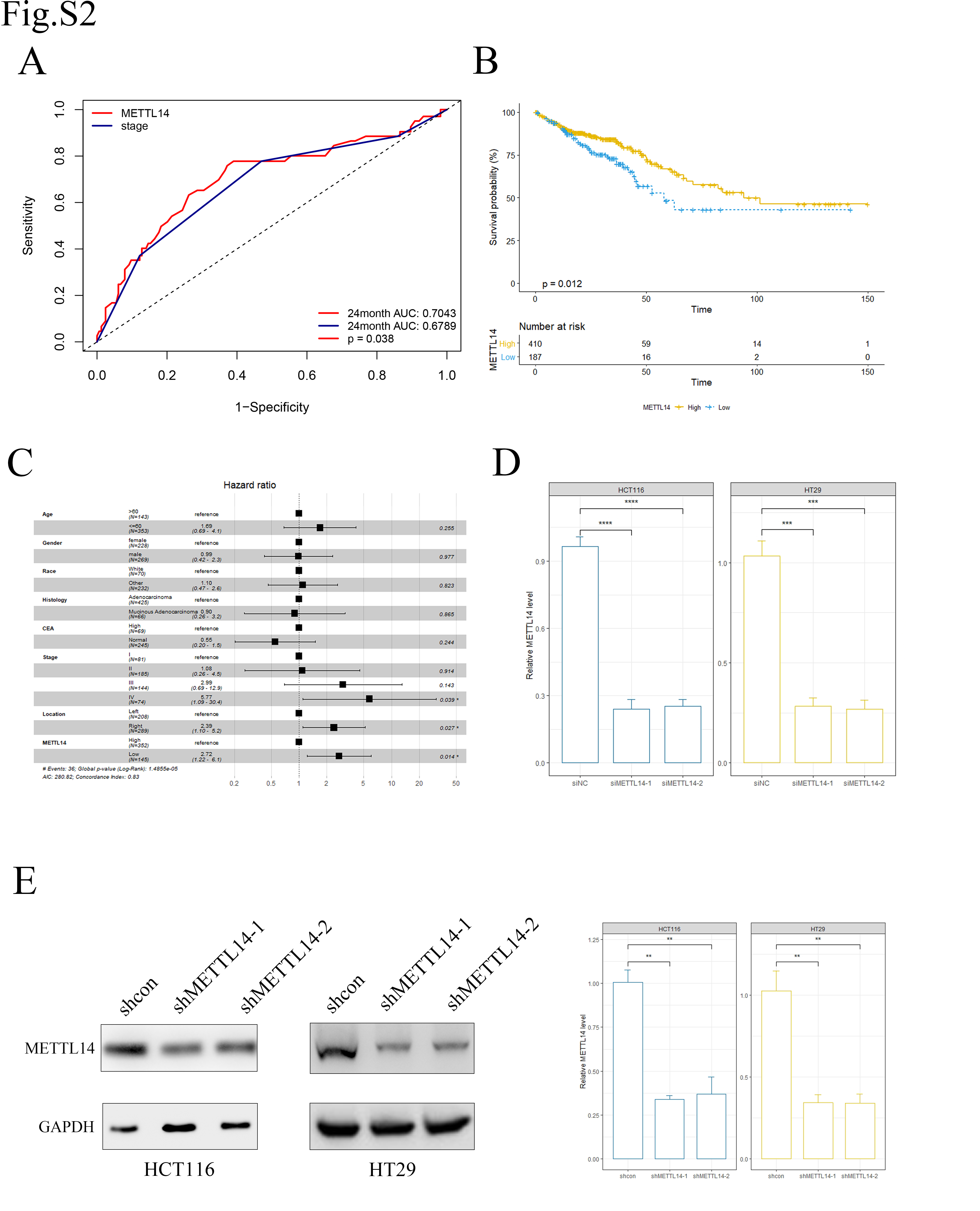

Supplement: Supplementary file 4 — Additional file 4: Figure S2. (A) Comparison of predictive ability of METTL14 and TNM stage of RFS of CRC patients at 24 months. AUC values were as shown. (B) CRC patients from TCGA colon adenocarcinoma (COAD) and rectal adenocarcinoma (READ) dataset were classified into METTL14-high and -low group and difference between two groups was compared with Kaplan-Meier analysis. (C) Multivariate analysis of TCGA data showed that METTL14 was an independent risk factor for OS of CRC patients. (D) Quantitative analysis of western blots of Fig.2a. (E) Knockdown of METTL14 was performed with two shRNAs and was confirmed by western blots. [file 12943_2020_1146_MOESM4_ESM.jpg]

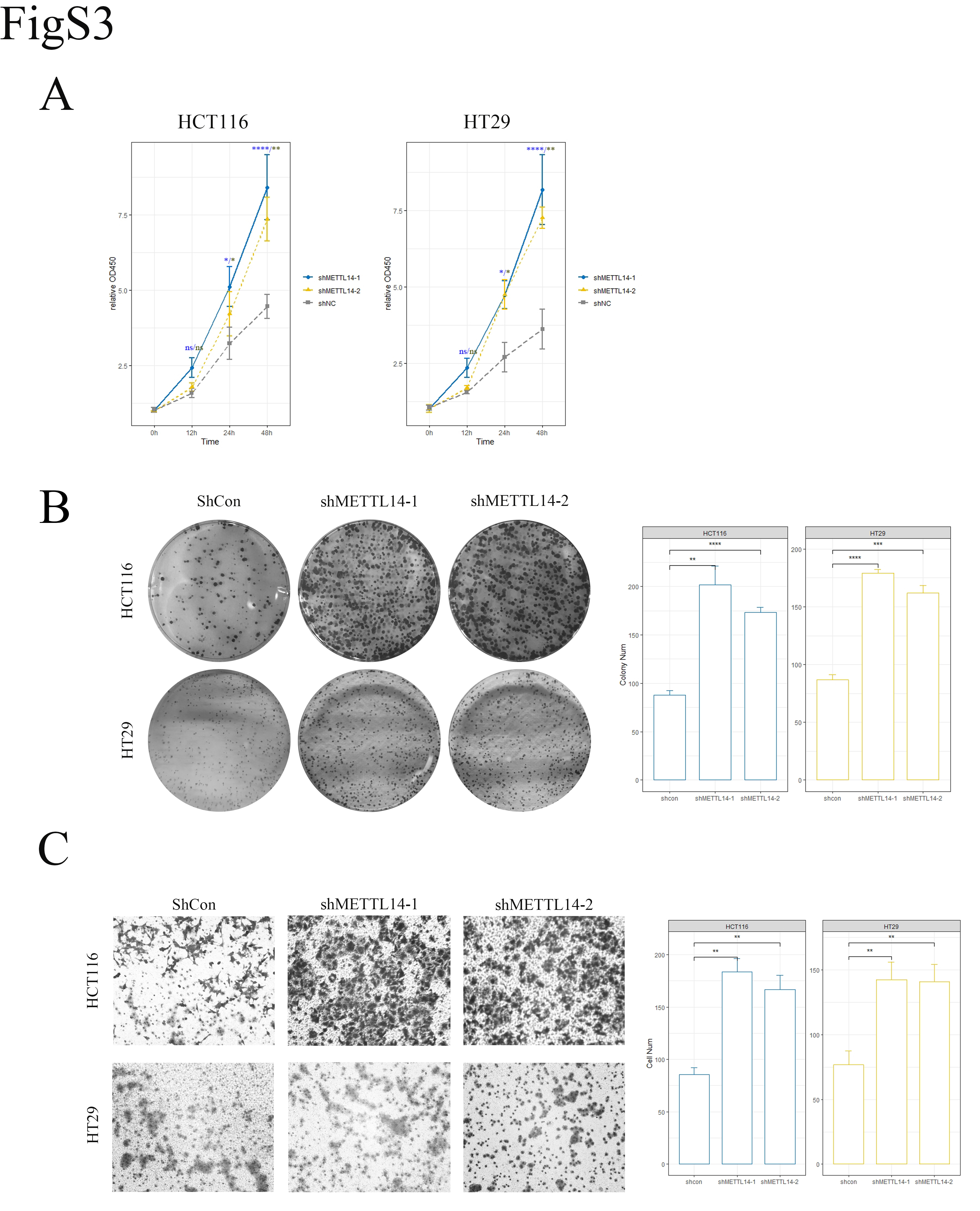

Supplement: Supplementary file 5 — Additional file 5: Figure S3. The proliferative and invasive ability of two shMETTL14 cells were tested by cell count assay (A), colony formation assay (B) and transwell assay (C). ns, not significant, *, p < 0.05, ****, p < 0.0001. [file 12943_2020_1146_MOESM5_ESM.jpg]

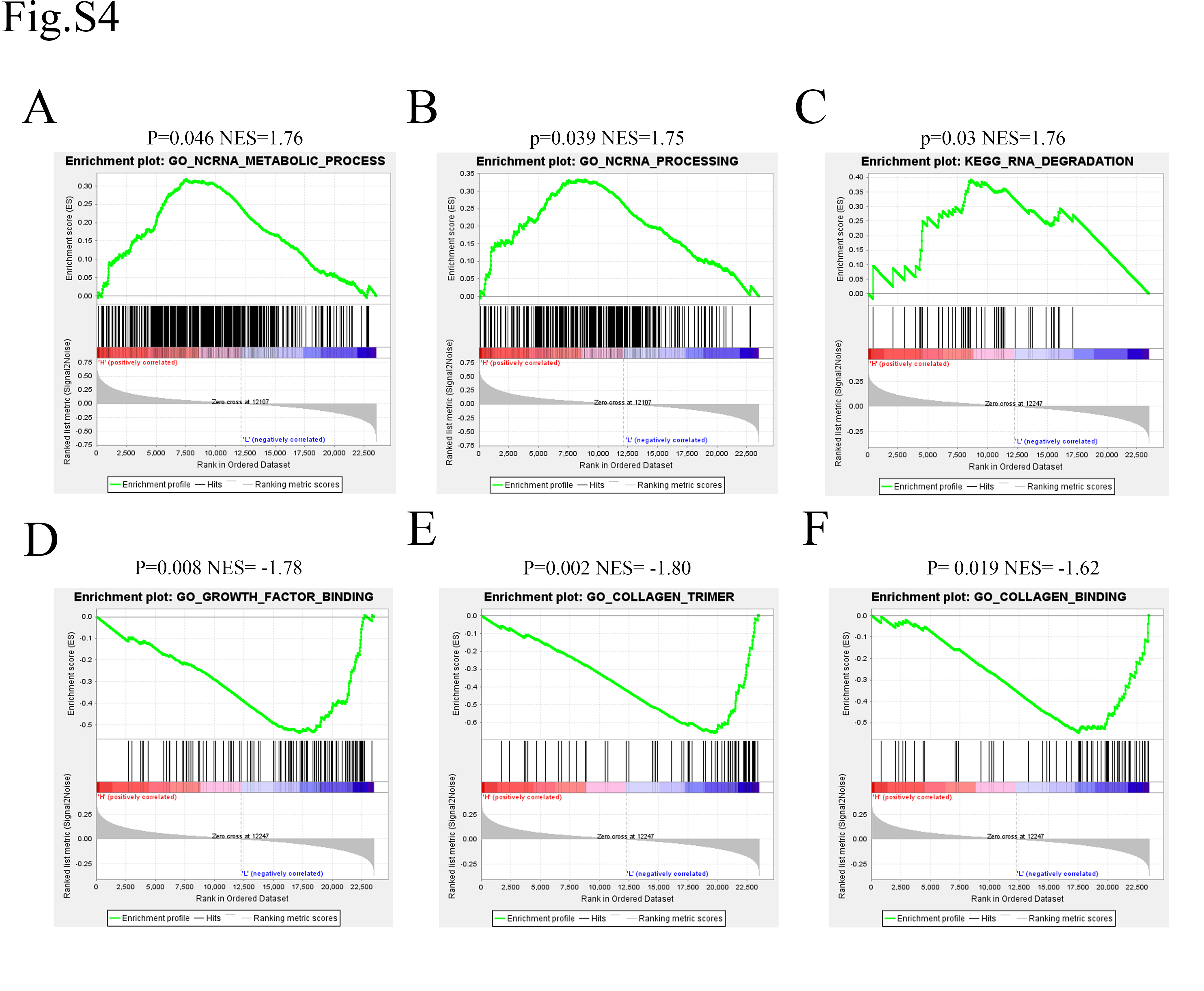

Supplement: Supplementary file 6 — Additional file 6: Figure S4. (A-C) METTL14 was positively correlated with regulation of non-coding RNA (ncRNA) metabolism (A), ncRNA processing (B), and RNA degradation (C). (D-F) METTL14 was negatively associated with growth factor binding (D), collagen trimer (E) and collagen binding (F). P values and normalized enrichment scores (NES) were as indicated. [file 12943_2020_1146_MOESM6_ESM.jpg]

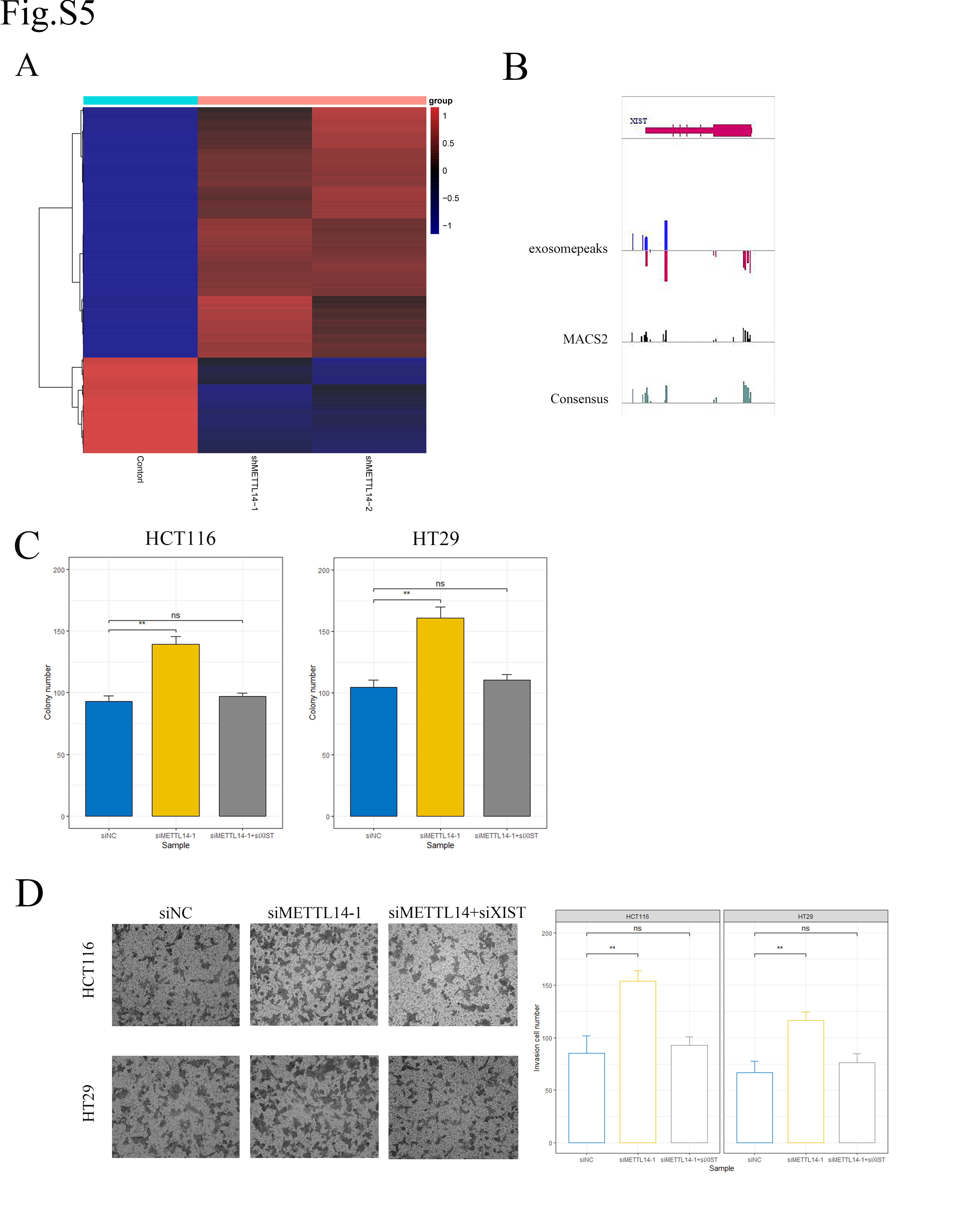

Supplement: Supplementary file 7 — Additional file 7: Figure S5. (A) Heatmap showed the DEGs between shMETTL14 and control cells. (B) Schematic diagram of m6A methylation sites of lncRNA XIST. Analysis was carried out by online tool MeT-DB (http://compgenomics.utsa.edu/methylation/). Potential m6A sites were shown as vertical bars. Three independent algorithms were applied. Details were shown in Additional file 1: Table S1. (C) Statistical histograms of Fig.4g. (D) Inhibition of XIST could attenuated the enhanced invasion of CRC cells resulted from METTL14 knockdown. [file 12943_2020_1146_MOESM7_ESM.jpg]

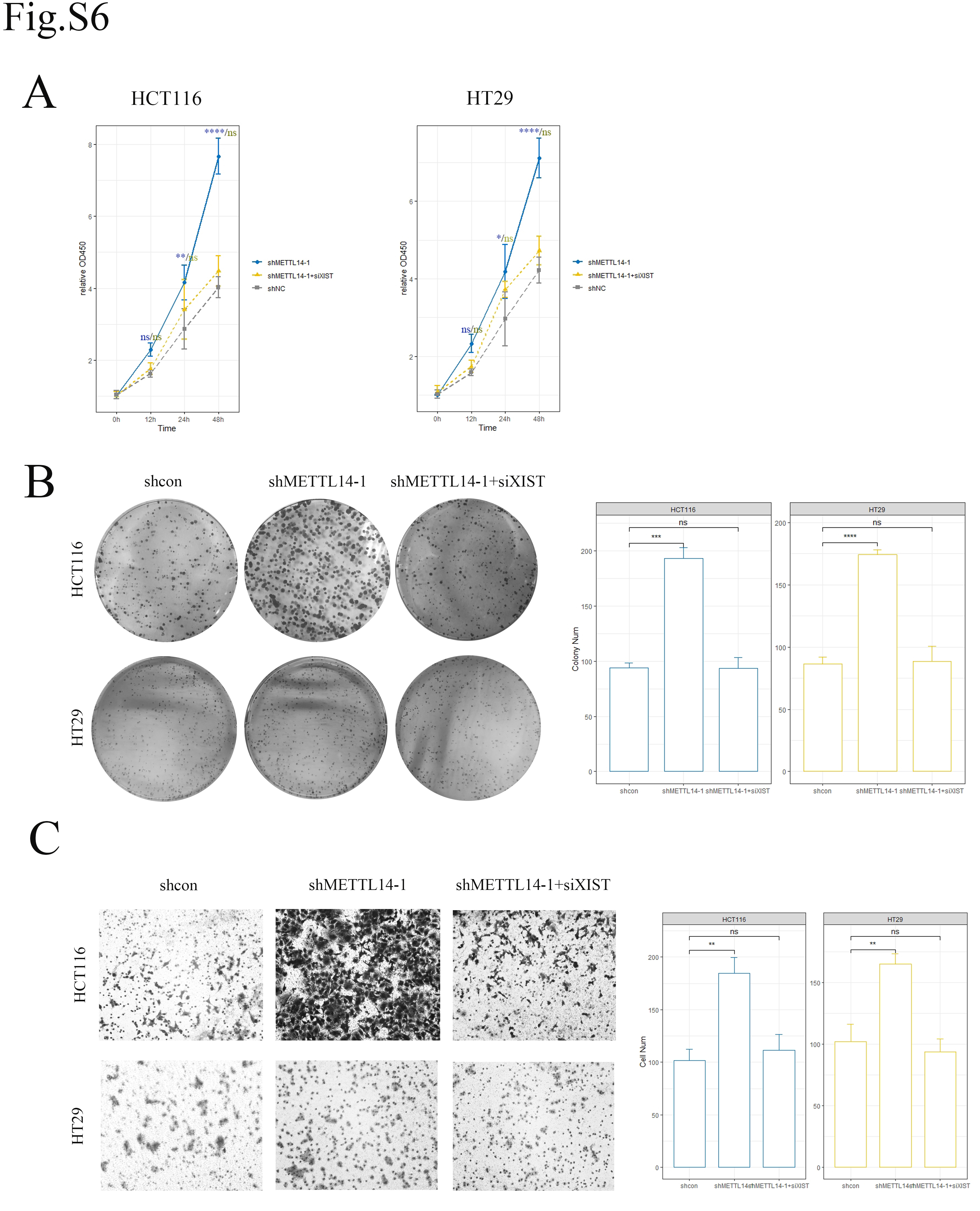

Supplement: Supplementary file 8 — Additional file 8: Figure S6. Inhibition of XIST was performed in shMETTL14 cells and the proliferative and invasive ability were then tested by cell count assay (A), colony formation assay (B), transwell invasion assay (C), respectively. The results showed that inhibition of XIST attenuated the increased cell growth and invasion of shMETTL14 cells. [file 12943_2020_1146_MOESM8_ESM.jpg]

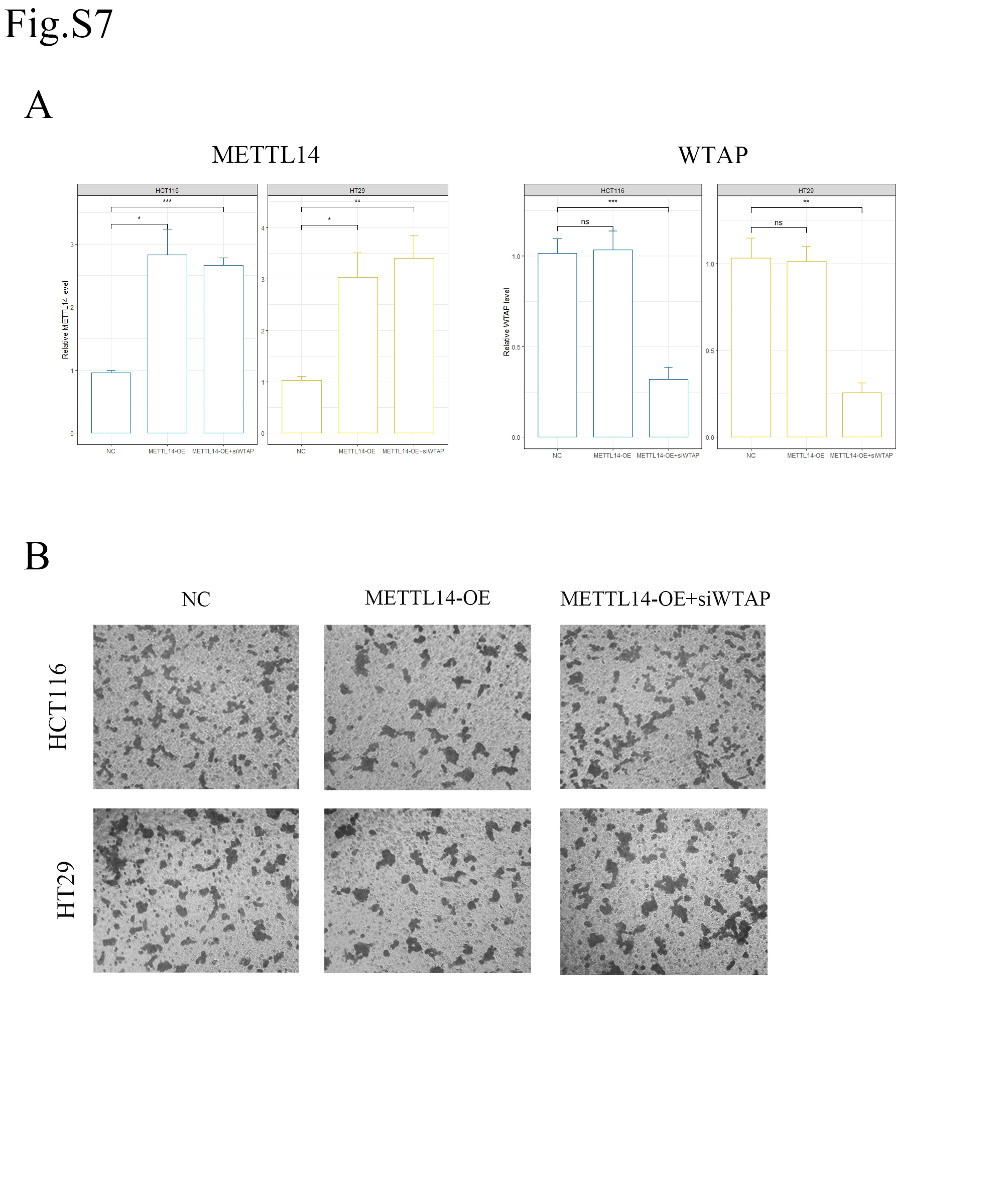

Supplement: Supplementary file 9 — Additional file 9: Figure S7. (A) Statistical analysis of protein bands from Fig.5a. (B) Representative images of transwell invasion assay of CRC cells with different expression status of METTL14 and WTAP. [file 12943_2020_1146_MOESM9_ESM.jpg]

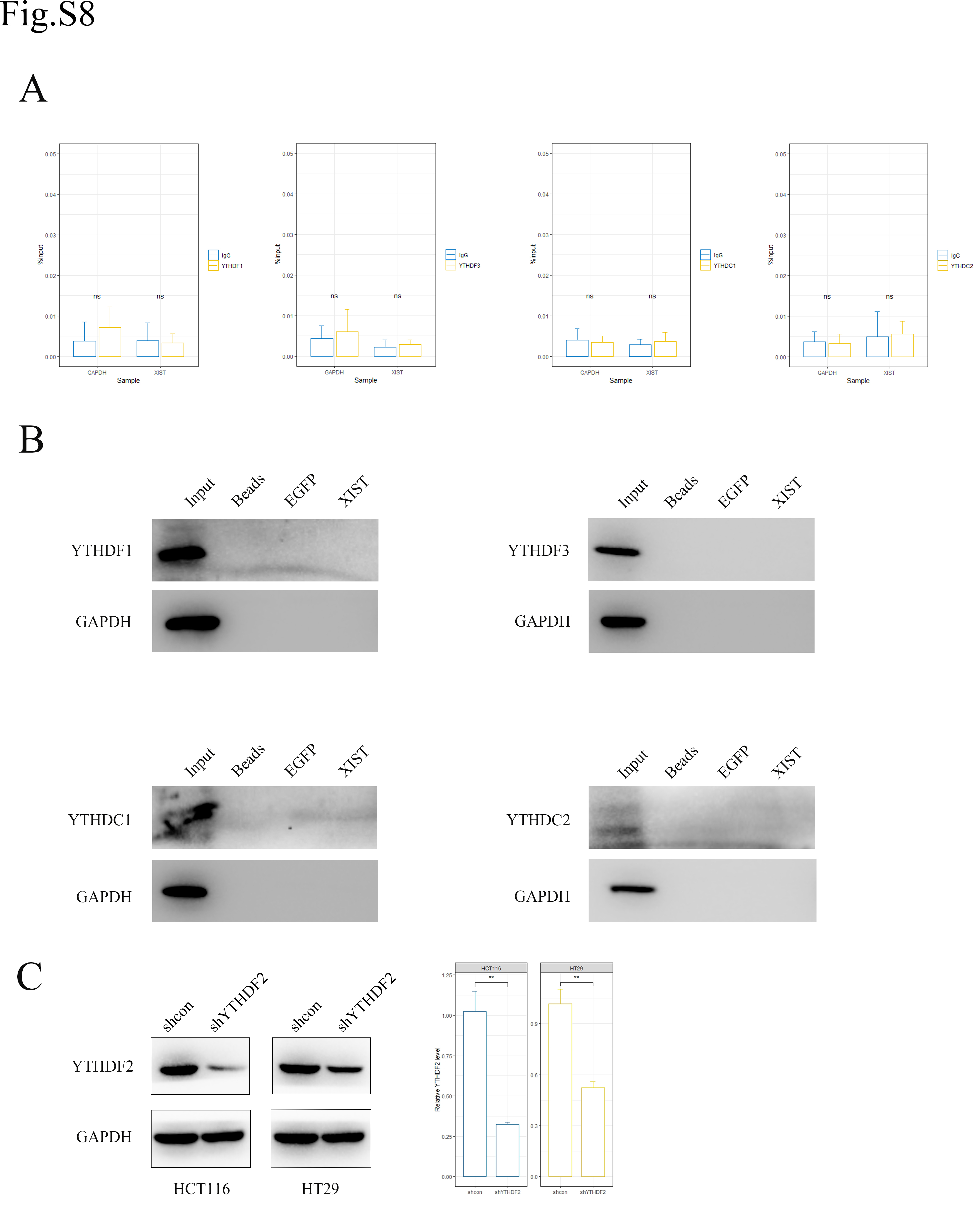

Supplement: Supplementary file 10 — Additional file 10: Figure S8. RIP (A) and RNA-pulldown (B) assay detected negligible binding between XIST and YTHDF1, YTHDF3, YTHDC1 or YTHDC2. ns, not significant. (C) Knockdown of YTHDF2 was confirmed with western bolts. [file 12943_2020_1146_MOESM10_ESM.jpg]
